# De Novo Evolutionary Emergence of a Symmetrical Protein Is Shaped by Folding Constraints

**DOI:** 10.1016/j.cell.2015.12.024

**Published:** 2016-01-28

**Authors:** Robert G. Smock, Itamar Yadid, Orly Dym, Jane Clarke, Dan S. Tawfik

**Affiliations:** 1 Department of Biological Chemistry, Weizmann Institute of Science, Rehovot 76100, Israel; 2 Metabolic Pathways and Enzyme Evolution Laboratory, Migal Galilee Research Institute, Kiryat Shmona 11016, Israel; 3 Department of Chemistry, University of Cambridge, Cambridge CB2 1EW, UK

## Abstract

Molecular evolution has focused on the divergence of molecular functions, yet we know little about how structurally distinct protein folds emerge de novo. We characterized the evolutionary trajectories and selection forces underlying emergence of β-propeller proteins, a globular and symmetric fold group with diverse functions. The identification of short propeller-like motifs (<50 amino acids) in natural genomes indicated that they expanded via tandem duplications to form extant propellers. We phylogenetically reconstructed 47-residue ancestral motifs that form five-bladed lectin propellers via oligomeric assembly. We demonstrate a functional trajectory of tandem duplications of these motifs leading to monomeric lectins. Foldability, i.e., higher efficiency of folding, was the main parameter leading to improved functionality along the entire evolutionary trajectory. However, folding constraints changed along the trajectory: initially, conflicts between monomer folding and oligomer assembly dominated, whereas subsequently, upon tandem duplication, tradeoffs between monomer stability and foldability took precedence.

## Highlights

• Inferred 47-aminoacid ancestral motifs fold into functional β-propeller assemblies
• Motif duplication, fusion, and diversification yield functional monomeric propellers
• Folding efficiency was the key parameter optimized throughout propeller emergence
• Single-motif precursors in extant genomes support the reconstructed emergence pathway

## Graphical Abstract

**Figure:**
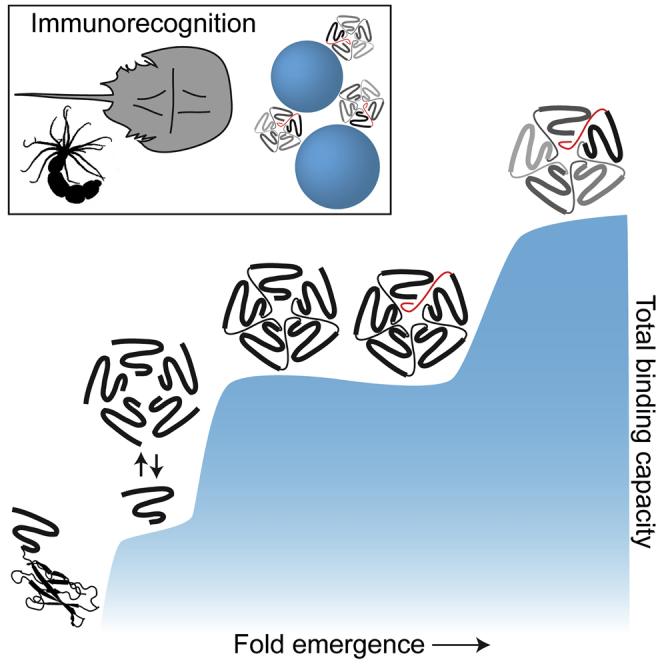

## Teaser

Experimental reconstruction of the emergence of a de novo protein indicates that “foldability” is the primary factor required for improved functionality along the entire evolutionary trajectory, although the parameters dictating optimal folding shifted as protein complexity increased.

## Introduction

The birth of new proteins is essential to the diversity of life, particularly in cellular signaling and immunity (Chen et al., 2013). Although global networks of relatedness among different protein superfamilies and folds have been derived (for a recent example, see Nepomnyachiy et al., 2014), an empirical description of the de novo emergence of a protein is lacking. A fuller understanding requires addressing the evolutionary intermediates leading to mature, fit proteins along with the molecular properties under selection.

**Figure 1:**
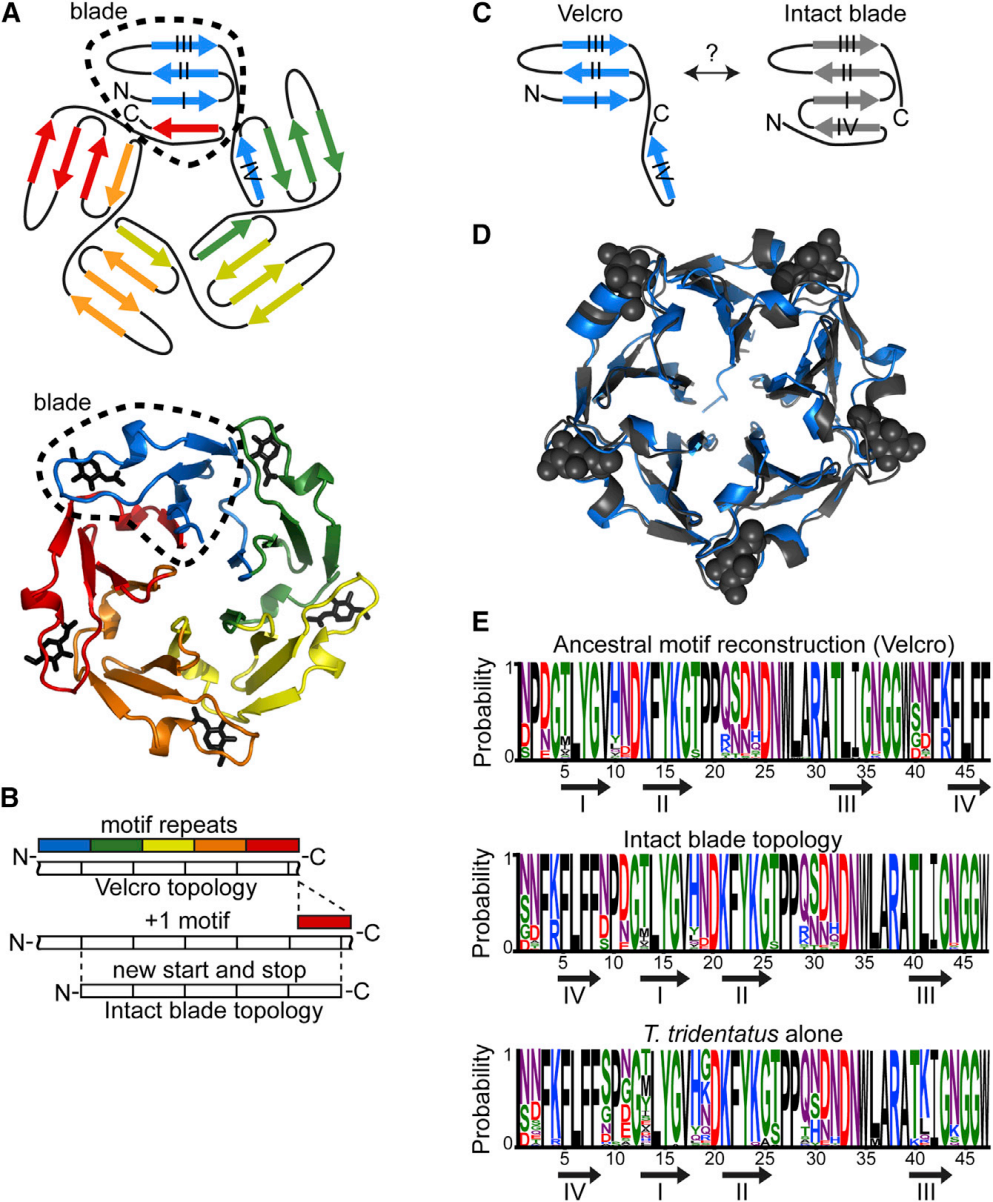
The β-Propeller Fold and Ancestral Motif Reconstruction (A) *T. tridentatus* tachylectin-2 is an extant lectin with the exemplary features of the propeller fold (PDB: 1TL2). It is composed of five sequence motifs, colored by order from blue (N terminus) to red (C terminus), comprising five structural motifs, or blades (in dotted outline). The five saccharide (GlcNAc in black sticks) binding sites are at the interfaces between the blades. Each motif and blade comprises four β strands (represented as arrows; numbered I–IV). However, one β strand is permuted so that the C-terminal strand IV completes the N-terminal blade to give the Velcro closure. (B) The genetic mechanism of topological permutation. Motif expansion followed by new start and stop codons allows interconversion between the Velcro closure and the intact blade topologies. The boundaries of blades (structural motifs) are shown in black and white. (C) Putative topologies of single motif proteins in the Velcro topology of extant tachylectin-2 and with permutation of strand IV to the topology of an intact structural blade. (D) Crystal structure of the homologous lectin, *N. vectensis* tachylectin-2 (determined at 1.9 Å; blue) superimposed with *T. tridentatus* tachylectin-2 (gray, PDB: 1TL2 with GlcNAc in spheres). The nearly identical structures share 44% sequence identity. (E) Reconstruction of the ancestral motif that gave rise to tachylectin-2 was performed by phylogenetic modeling of the 10 *T. tridentatus* and *N. vectensis* motifs. FastML gave a probabilistic model of amino acid states for the single ancestral motif (top). The intact blade permutation frame is also shown (middle). The inference did not significantly differ when based on the motifs of *T. tridentatus* alone (bottom). See also Figure S1.

Duplication and fusion of a short ancestral motif underlie the structural symmetry so commonly observed in modern proteins (Balaji, 2015). Accordingly, we define a “motif” as the smallest repetitive sequence unit that a symmetric, globular protein can be broken into. Symmetry is particularly dominant in all β proteins, and distinctly in β propellers (Balaji, 2015). β Propellers are associated with diverse functions in immunorecognition, viral infection, signal transduction, and vesicle formation. Propellers comprise four to eight blades arranged in radial symmetry, with each blade comprising a four-strand β sheet (Figure 1A). The repeated β-sheet motif shares homology with other fold groups, thus suggesting multiple, parallel emergence events from short peptide motifs (Kopec and Lupas, 2014). Tandem sequence repetitions are also frequently observed in genomes (Verstrepen et al., 2005). Thus, emergence by co-option of a short sequence motif, followed by duplication and fusion of the DNA segment encoding this motif, is genetically feasible. Topological permutations are also a commonly observed genetic rearrangement, involving duplication, fusion, and truncation (new start and stop codons) that effectively transpose residues between protein termini (“circular permutation”; Figures 1B and 1C). These permutations shift the boundaries between sequence motifs and structural domains (Longo et al., 2013, Peisajovich et al., 2006), as seen in the “Velcro closure” topology of propellers (Figures 1A–1C). However, for these mechanisms to be evolutionary feasible, the genetic processes generating new DNA sequences must be coupled with protein intermediates that are foldable, stable, and biochemically active.

**Figure 2:**
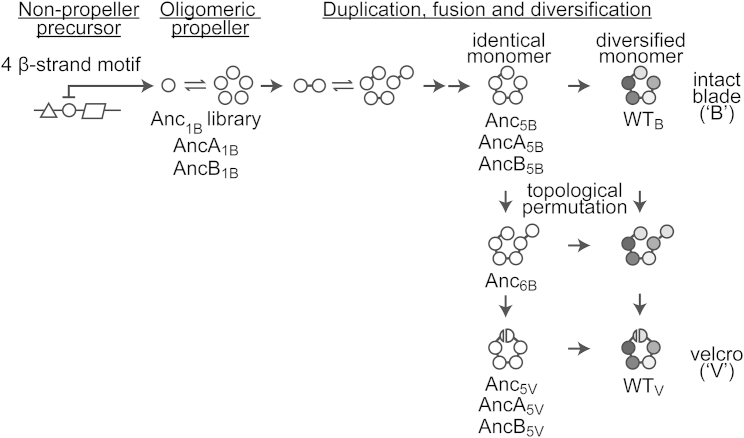
De Novo Emergence and Maturation of Ancestral Motifs A basic evolutionary trajectory leading to tachylectin-2 is illustrated. The emergence of WT tachyelctin-2 from single ancestral motifs is likely promoted by genetic mechanisms that include gene duplication, fusion, diversification, and topological permutation. Key experimental constructs are annotated in this scheme. For simplicity, alternative intermediates such as fusions of two to four motifs and their diversification and fusion to non-propeller domains are not depicted. WT stands for sequence of the extant, WT tachylectin-2. Anc stands for reconstructed ancestral variants, as single motifs (Anc_1_) or duplicated tandem fusions (Anc_5_); V subscripts indicate the extant Velcro topology, whereas B subscripts represent the alternative intact blade topology.

Experimental reconstruction of symmetric proteins has gained attention as an evolutionary model and a design paradigm (Höcker, 2014, Longo and Blaber, 2014, Park et al., 2015). For example, a designed single 42-amino-acid β-trefoil motif assembled as homotrimer, albeit with no function (Lee and Blaber, 2011). Functional six-bladed propellers were assembled from designed two or three-motif fusions (Voet et al., 2014). Conversely, function was observed in fused monomers that are identical or nearly identical, yet with no self-assembly from single motifs (Broom et al., 2012, Claren et al., 2009, Yadid and Tawfik, 2011). However, one crucially missing link is a single-motif ancestor inferred from an extant symmetric protein that is capable of self-assembly into a biochemically active protein. Such single motifs have not yet been reconstructed in the laboratory or observed in nature, and we lack a description of a viable evolutionary trajectory leading from an ancestral single motif to an extant symmetrical protein through a series of functional intermediates (Figure 2).

Here, we address a basic evolutionary trajectory leading to an existing, natural lectin β propeller. First, we show that single ancestral motifs inferred from the sequence repeats of the extant propeller yield a functional lectin via oligomerization. We subsequently examined how these motifs further evolve by tandem duplications, diversification, and topological permutation to yield highly functional monomeric lectins (Figure 2). An obstacle related to the evolution of repeating domains in tandem arrays (“beads on a string”) is that high internal sequence identity promotes aggregation via domain-swapped misfolding, suggesting that sequence diversification is crucial for efficient folding (Borgia et al., 2011, Wright et al., 2005). Whether folding restricts the functionality of globular repeat proteins such as propellers and how stability and foldability evolve need to be explored (Balaji, 2015, Zheng et al., 2013). We thus examined the biophysical features that underlie the evolutionary optimization of the newly emerging lectin propellers starting from a single motif.

Our results validate the hypothesized mechanism for the de novo emergence of a functional lectin β propeller from short motifs (<50 amino acids). A selectable function was demonstrated for the proposed intermediates along the basic trajectory, with gradual functional improvement along a genetically feasible pathway of tandem duplication and repeat diversification, both via point mutations and frame permutations, leading to the extant lectin β propeller. The biophysical properties of various constructs were analyzed, thus offering a glimpse of the features shaping the evolution in terms of binding affinity, configurational stability, and folding efficiency. Emergence from an ancestral single motif that duplicates, fuses, and diverges was also supported by identifying natural genes containing single propeller motifs that were related to mature propellers in the same genome.

## Results

### Phylogenetic Inference of Ancestral Motifs

Although propellers were hypothesized to have emerged by duplication and fusion of short sequence motifs (Vellieux et al., 1989), high internal identity is rarely preserved (Wright et al., 2005). Nonetheless, because of the ubiquity of the propeller fold, of 95,306 non-redundant propeller sequences identified in the Pfam database, nearly 1,000 sequences exhibited ≥50% average internal identity. Among these, tachylectin-2 from the horseshoe crab *T. tridentatus* provides an excellent model for the study of propeller evolution. First, the approximation of an ancestral motif is facilitated by relatively little divergence among its five tandemly repeating motifs (54% average internal identity). Second, tachylectin-2 is an immunoprotein that agglutinates foreign cells by multivalent binding of surface GlcNAc- and GalNAc-decorated glycoproteins. Its physiological role is utterly dependent on multivalent binding, suggesting that internal symmetry and the comparatively high internal sequence identity are directly linked to function (Balaji, 2015). The repeating motifs encode both the globular propeller fold and the rigid binding of saccharides between propeller blades (Figure 1A).

**Figure 3:**
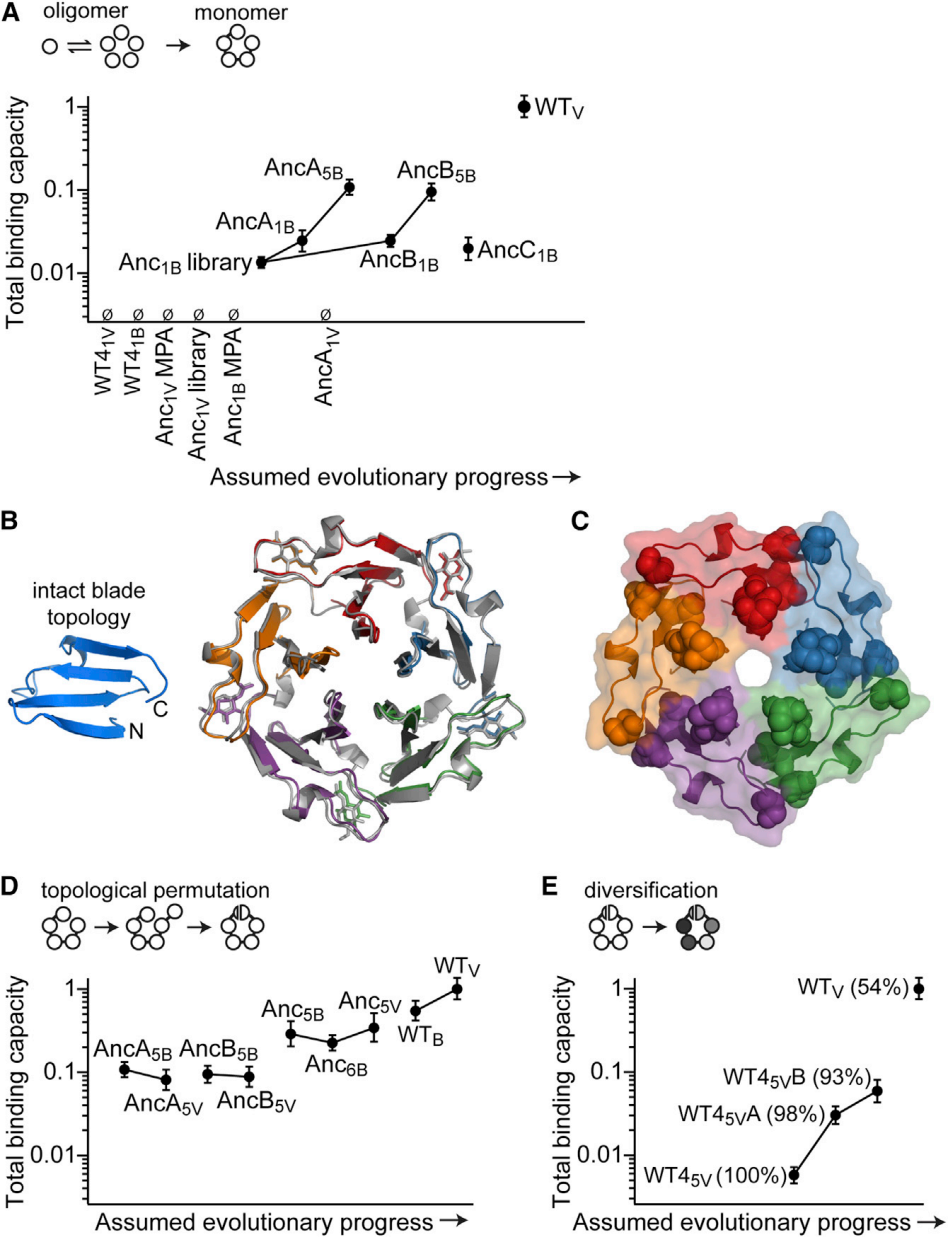
Ancestral Single Motifs Are Functional as Oligomers and in Tandem Fusions The total binding capacity of single motif lectins was measured by the mucin-binding signal of cell lysates in ELISA. The null sign (Ø) indicates no detectable binding. (A) The early steps of the presumed trajectory depicted in Figure 2. Functional single motifs were isolated from ancestral substitution libraries in the permutation frame of an intact blade (Anc_1B_ library). WT-derived motifs (WT4_1V_, WT4_1B_), a single MPA motif (Anc_1B_ MPA), and ancestral libraries in the Velcro frame (Anc_1V_ library) were non-functional. Identical tandem fusion of the functional single motifs (e.g., AncA_1B_ to AncA_5B_ and AncB_1B_ to AncB_5B_) enhanced total binding capacity with no other change in sequence. (B) The crystal structure of AncB_1B_ bound to GlcNAc (1.7 Å) revealed a pentamer (five colored subunits) in a permuted topology that is nearly identical to the WT tachylectin-2 monomer (gray, PDB: 1TL2). (C) Crystal structure of the single, oligomerizing motif AncB_1B_. Mutations that converged in the selection of single motifs localized along subunit interfaces and near the GlcNAc binding site (spheres: N12D and K42L from the first ancestral library; T14M and S31N from the second ancestral library; F23L from error-prone PCR). (D) In later steps of the possible trajectory, topological permutations of tandem fusions led to the extant Velcro frame with relatively little consequence in total binding capacity (e.g., Anc_5V_ derived from Anc_5B_). The putative intermediates enabling these permutations (Anc_6B_) were also viable. All sequences except WT_V_ and WT_B_ comprise internally identical motif repeats. (E) The total binding capacity of an identical tandem fusion of the WT fourth motif (WT4_5V_) was improved by selective diversification following random mutagenesis. Internal sequence identity is shown in parentheses. See also Figures S2 and S3 and Table S1.

Many potential trajectories that lead to tachylectin-2 can be envisioned in sequence, structure, and function. As a starting point, however, we focused our investigation on the most basic trajectory depicted in Figure 2, with a single motif that forms a functional pentamer being the key, founding step. However, the smallest fragments of extant tachylectin-2 that gave a functional propeller consisted of two repeats (∼100 amino acids) (Yadid and Tawfik, 2007). Further, the fourth motif of WT tachylectin-2 that best represents the consensus sequence (Yadid and Tawfik, 2011) did not show a detectable lectin function as a single motif, in either the Velcro (WT4_1V_) or the intact-blade permutation frame (WT4_1B_), and its tandem fusion showed very low binding capability (Figures 3A and 3D). Ancestral properties, in this case the presumed ability of a single motif to self-assemble into a functional lectin, are often lost in extant sequences through mutational drift that blocks reversion to the ancestral features (Afriat-Jurnou et al., 2012, Bridgham et al., 2009). We therefore aimed to capture the potential for emergence from a functional single motif by ancestral inference.

The computational inference of ancestral sequences aims to identify the most probable sequences from which a given set of extant sequences, relating to one another via a given phylogenetic tree, diverged. Being based on generic probabilities of amino acids exchanges (substitution matrixes), inference is statistical, not deterministic (Merkl and Sterner, 2015). Thus, per each position, sets of amino acids are predicted, each with a given probability. The probabilities depend on the substitution matrix, the number of available extant sequences, their identity level, and the consistency of their phylogenetic relationships. The most probable inferred ancestor (MPA) relates to a sequence in which all positions comprise the amino acid predicted with the highest probability. However, the MPA is just one sequence from an entire “cloud” of sequences that are in effect as probable.

**Figure S1:**
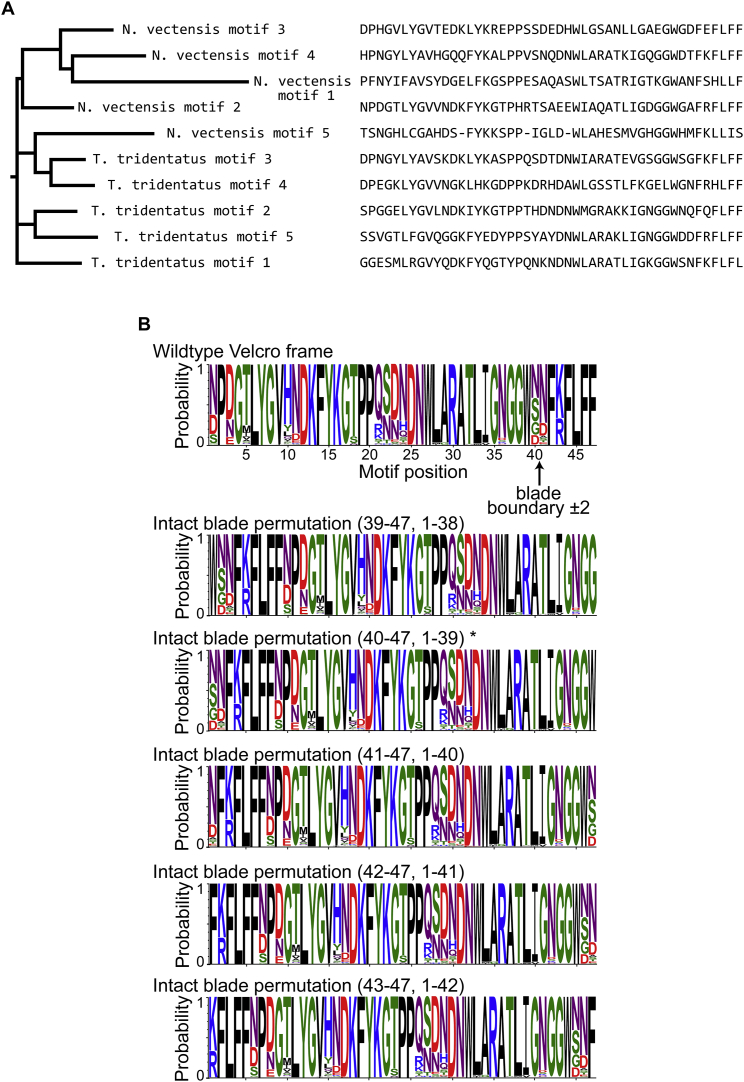
Ancestral Reconstruction of the Lectin Propeller Motif, Related to Figure 1 (A) A phylogenetic tree of tachylectin-2 sequence motifs was constructed from an alignment of ten individual motifs of the *T. tridentatus* and *N. vectensis* tachylectin-2 sequences. Excluding *N. vectensis* motif 5, the motifs are monophyletic with respect to the source protein. The tree topology could not be improved by inclusion of an *X. laevis* outgroup sequence. (B) A probabilistic ancestral motif was reconstructed from the root node of the motif tree in (B). The posterior probabilities of amino acids given by FastML are plotted as sequence logos in the WT Velcro frame and in five additional permutation frames corresponding to an intact structural blade that were tested experimentally. An asterisk (^∗^) indicates the frame from which functional single motifs were obtained.

To infer the ancestral motif from which tachylectin-2 may have diverged, we separated and aligned the five-sequence motifs of tachylectin-2 from *T. tridentatus.* Tachylectin-2 is a near-orphan protein, but we identified a sea anemone homolog which is 44% identical in sequence and essentially identical in structure and function (Figure 1D). The aligned individual motifs were assembled in a phylogenetic tree (Figure S1A) and the common ancestral motif was inferred (Figures 1E). The caveats associated with this inference are a limited number of sequences and unknown phylogeny (the species where the two lectins are found are not closely related). However, because of the high sequence identity and unambiguous alignment, the prediction seems comparatively robust (34 of 47 positions predicted with ≥90% probability). Further, the ancestral sequence inferred from only *T. tridentatus* tachylectin-2 motifs was essentially identical (Figure 1E), thus indicating that inference is independent of the two lectins sharing a common ancestor.

Given the statistical nature of ancestral inference, the reconstructed ancestor is best represented by a library of sequences with combinatorial sampling of alternative predictions (Bar-Rogovsky et al., 2015). Such libraries were constructed here, thus testing all combinations of the alternative states (>25% probability) in the MPA’s background. The library was also extended to include different permutation frames beyond the Velcro topology of extant tachylectin-2. The alternative frames relate to the β strands topology in tachylectin-2, such that intact structural blades could be obtained with “end polishing” of ±2 residues (Figures 1C, 1E, and S1B). Overall, the first single motif library included 23 natural amino acid substitutions at ten positions within six different permutation frames (∼6 × 10^3^ variants).

### The Emergence of Functional Single Motifs

**Figure S2:**
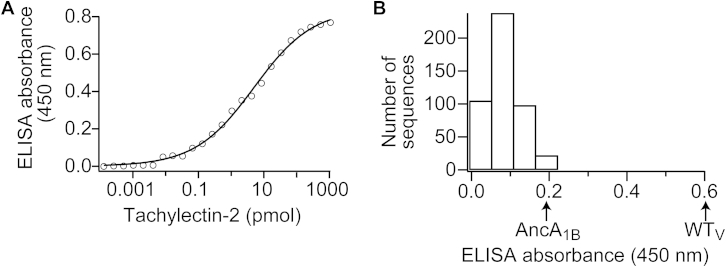
Correspondence of ELISA Signal to Binding Activity and the Variety of Functional Single Motifs, Related to Figure 3 (A) To calibrate the non-linear response of ELISA to the functional level of tachylectin-2, raw ELISA signals were measured from a purified WT tachylectin-2 concentration gradient. Data were fit to the sigmoidal function $y=0.843/\left(1+{\left(5.00/x\right)}^{0.483}\right)$ whereby *y* is the measured absorbance and x is tachylectin-2 concentration. (B) Single motifs containing the most probable ancestral substitutions (second round library, p > 0.1) were constructed as a mixture of plasmids (∼10^4^ single motif sequences). This library was transformed into *E. coli* and sampled from the cell lysates of 500 randomly chosen clones. Raw ELISA absorbance was subtracted with the background of an empty plasmid’s expression lysate.

To estimate the “fitness” of the ancestral motifs and all subsequent intermediates along the trajectory, the lectin binding capacity was measured in crude lysates of *E. coli* cells in which these constructs were overexpressed. The *total binding capacity* of cell lysate, expressed in arbitrary, relative units (see Experimental Procedures), reflects both the level of properly folded and functional protein and the specific activity, i.e., the affinity for glycoprotein. Specifically, we measured binding to a glycoprotein, mucin, using an ELISA format whereby lectin binding to immobilized mucin was determined with polyclonal anti-tachylectin-2 antibodies. This assay exhibited high sensitivity and a wide dynamic range (Figure S2A). The more physiologically relevant binding of saccharide-decorated cell surfaces was also measured by hemagglutination and was found to corroborate the ELISA data (Table S1).

Single motifs isolated from the ancestral library that corresponded to an intact blade frame encoded functional lectins (Anc_1B_ library; Figure 3A) while no function was observed with the extant Velcro frame (Anc_1V_ library; the nomenclature of constructs is described in the legend to Figure 2). Thus, it appears that topological permutation(s) occurred only at later stages of the trajectory leading to tachylectin-2 (Figure 2). Additionally, while the exact MPA blade gave no binding (Anc_1B_ MPA; Figure 3A), several library variants with the blade frame were functional. Thus, the uncertainties associated with ancestral inference are best tackled via a library approach. The functional signal associated with these ancestral motifs was low (2% relative to WT). Nonetheless, hemagglutination could be observed at concentrations of ≥10 nM (Table S1), suggesting that given high enough expression levels these short motifs would be functional in vivo.

A second-generation library was constructed based on the first generation’s most active variants. Amino acids that were enriched to convergence in the first generation were fixed, and new ancestral inference alternatives were introduced at additional positions (>10% probability). Screening of the second library gave hundreds of functional motifs (Figure S2B). The best performing single motif in this library, AncA_1B_, showed 2-fold higher total binding capacity compared with the first generation (Figure 3A). As observed with the first generation (Anc_1B_ library), topological permutation of AncA_1B_ to a Velcro frame (AncA_1V_) resulted in an inactive protein, again highlighting the critical dependency of the single motif on an intact blade topology at this early evolutionary stage.

We also tested the viability of single motif sequences that further deviate from the MPA. The best performing sequence from the first round of selection (Anc_1B_ library) was subjected to error-prone PCR at a rate of 1.6 mutations per motif. Selection identified a single mutation, F23L (AncB_1B_), which was incidentally an ancestral state predicted with low probability; this mutant exhibited similar total binding capacity to AncA_1B_. Accordingly, introducing F23L into AncA_1B_ (AncC_1B_) did not alter the total binding capacity, suggesting a certain level of sequence redundancy (Figure 3A). Overall, the library selections indicate the high probability of emergence of functional ancestral single motif(s) that, jointly, form an entire “cloud” of functional sequences that relate with high probability to the MPA, even if the MPA itself is non-functional.

### Single Motifs Assemble as Homo-pentamers

**Figure S3:**
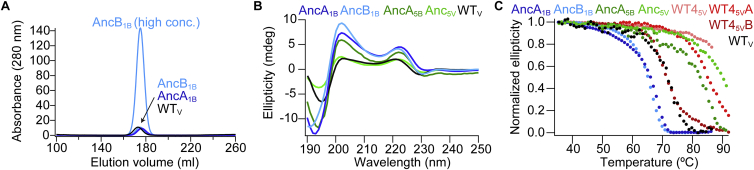
Native Propeller Formation and Stability, Related to Figures 3 and 5 (A) Size exclusion chromatography revealed monodisperse and overlapping elution profiles for tandem fusions and single motif constructs, indicating stable pentamerization of the latter even at low concentration. The upper AncB_1B_ trace is at high concentration (12 μM propeller) and lower traces are at low concentrations (0.8 μM propeller). (B) Proteins showed similar propeller-like signatures by circular dichroism (CD). Shown are native CD spectra measured at 30°C. (C) Thermal unfolding was measured by CD (202 nm). In cases of unresolved baselines at high temperature, normalization to zero was assisted by measuring the residual function in ELISA.

AncA_1B_ and AncB_1B_ were purified with a sugar ligand (GlcNAc) resin and eluted indistinguishably from WT tachylectin-2 in gel filtration, indicating the formation of stable pentamers; additional peaks corresponding to alternate states were not observed (Figure S3A). Circular dichroism (CD) spectra also had similar curve shapes to the WT spectrum (Figure S3B). The crystal structure of AncB_1B_ was essentially identical to the WT structure despite its alternate permutation frame, with each of the pentameric subunits cradling GlcNAc in the same binding orientation as the five-bladed WT monomer (Figure 3B). The repetitively observed mutations of selected single motifs localized along the subunit interfaces and near the GlcNAc binding sites (Figure 3C).

### Maturation by Duplication, Fusion, and Diversification

The next step in the basic trajectory is duplication and fusion in tandem. To reconstruct this step, each of the single motifs AncA_1B_ and AncB_1B_ were identically duplicated 5-fold and fused in tandem to give AncA_5B_ and AncB_5B_, respectively (Figure 2). The duplication-fusion step gave a 4-fold increase in total binding capacity in both cases (Figure 3A). Like-wise, tandem fusion of Anc_1B_ gave the functional Anc_5B_. This result further validates the ancestral inference, also in light of the tandem fusion of WT fourth motif, WT4_5V_, being barely functional. Thus, while the pentameric single motif proteins are functional, there is a clear selective advantage associated with duplication and fusion into a multi-motif, monomeric protein.

The single motifs were only functional with a blade topology (Figure 3A; Anc_1B_ library versus Anc_1V_ library and AncA_1B_ versus AncA_1V_), indicating that the Velcro topology seen in tachylectin-2 and most other propellers emerged at a later stage. Indeed, while the intact blade frame of the single motifs seems obligatory, following duplication and fusion, permutation had a surprisingly modest effect on total binding capacity. Motif expansion of Anc_5B_ gave a functional 6-motif protein, namely Anc_6B_, as did truncation of Anc_6B_ to give the five-motif protein in the new Velcro topology, namely Anc_5V_ (Figure 3D). Similar observations were made with the other duplicated-fused intermediates in the alternate frames (AncA_5B_ to AncA_5V_ and AncB_5B_ to AncB_5V_). Accordingly, permutation of WT tachylectin-2 from the extant Velcro frame (WT_V_) to the ancestral blade frame (WT_B_) resulted in a less than 2-fold decrease in total binding capacity (Figure 3D).

As mentioned above, the extant tachylectin-2 sequence lost the ancestral capability to function as a single motif and was also severely impaired as a tandem fusion (WT4_5V_; Figure 3E). Diversification of WT4_5V_ via the introduction of alternative WT motifs previously led to functional lectins, but these constructs diverged from WT4_5V_ at >20 positions (Yadid and Tawfik, 2011). A more feasible route to diversifying selection was therefore explored by random mutagenesis of WT4_5V_ using error-prone PCR at a rate of approximately three mutations per gene. Indeed, improvements in total binding capacity were observed in response to few mutations: WT4_5V_A in the first round and WT4_5V_B in the second (Figure 3E). The F23L mutation in the single motif AncB_1B_ also featured in the evolving WT4_5V_ with F-to-L mutations at equivalent positions (WT4_5V_A and WT4_5V_B were each substituted with F23L of their second and fourth repeats, among other mutations).

While diversification plays a role in selection, interestingly, constructs with identical repeats had total binding capacities covering a range of a 100-fold (compare AncA_5V_, AncB_5V_, Anc_5V_, and WT4_5V_ in Figures 3D and 3E). Thus, the magnitude of selection toward repeat diversification (lower internal sequence identity) was highly dependent on the repeat sequence. The identical fusion WT4_5V_ sequence became more functional upon mutation and selection. However, even when its internal identity decreased to 93%, it was still inferior relative to the fully identical ancestral fusions. This gave another indication that the ancestral reconstruction was relevant and that the ancestral trait of a single, oligomerizing motif that subsequently duplicated and fused was lost in extant tachylectin-2.

### Foldability Is the Main Parameter under Selection

Having reconstructed a possible scenario for the de novo emergence of a lectin β propeller (Figure 2), we next sought to disentangle the different biophysical properties that underlie the total binding capacity of the various evolutionary intermediates (Figure 3). The earliest evolutionary stage of single motifs was sampled using AncA_1B_ and AncB_1B_; identical fusions were sampled using AncA_5B_ and Anc_5V_, and WT_V_ represented the extant diversified fusion. First, we measured the levels of soluble protein following expression and cell lysis and of aggregated protein in the insoluble pellet. These describe the amounts of natively folded versus aggregated protein. They relate to the efficiency of folding of individual variants, but also to cellular factors such as chaperones and proteases (Bershtein et al., 2013, Hingorani and Gierasch, 2014). Next, three biophysical properties of the purified, folded proteins were investigated. Specific activity usually dominates the divergence of new proteins, and therefore, GlcNAc binding affinity was measured. Folding efficiency was also measured in vitro, in the absence of cellular factors, by following the residual binding function (ELISA signal) after chemical denaturation and renaturation by dilution into buffer. Finally, stability of the native, folded state may also dictate the levels of functional protein, as in the cellular milieu, unstable folded states may result in misfolding, proteolysis, and/or aggregation. The stability of the native state was measured by the global unfolding midpoint in CD thermal melts.

**Figure 4:**
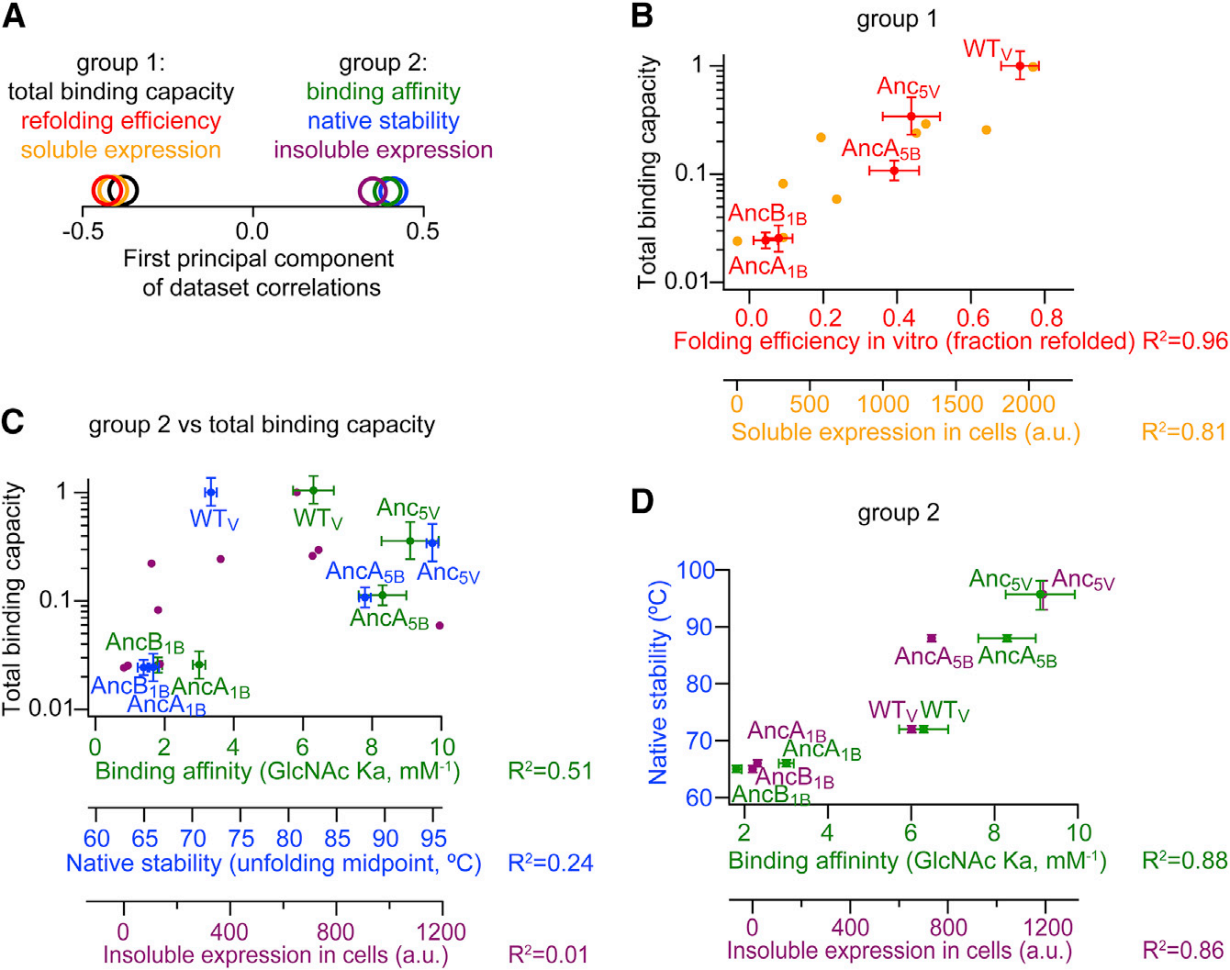
Folding Efficiency Was the Primary Trait under Optimization (A) In addition to total binding capacity (Figure 3), five different parameters were measured for representative variants along the evolutionary trajectory: the levels of soluble protein in crude cell lysates, the level of insoluble aggregates, binding affinity to GlcNAc, thermal stability of the native state, in vitro folding efficiency. Correlations between the six datasets, as indicated by groups 1 and 2, were detected by principal components analysis applied without prior bias. (B) As indicated in (A), folding efficiency in vitro and soluble expression in vivo were highly correlated with each other and with total binding capacity in cell lysates. The R-squared linear regression statistic is shown on each x axis. (C) Native stability, binding affinity, and the levels of insoluble aggregates were poorly correlated with total binding capacity overall, but did show a consistent trend by which identical fusions (AncA_5B_ and Anc_5V_) showed the highest values for each of these properties. (D) Native stability, binding affinity and the levels of insoluble aggregates were highly correlated to each other. See also Table S2.

The relationships between these five measured parameters and between these parameters and the total binding capacity were examined by an unbiased regression analysis. A 6 × 6 correlation matrix was constructed (Table S2) and sorted by its principal components. The first principal component explained 85% of the variance among all intercorrelations and indicated two groups of correlated parameters (Figure 4A). Group 1 included total binding capacity and folding efficiency, as reflected by the levels of soluble protein in *E. coli* and/or by the yield of folding in vitro (Figure 4B). In contrast, affinity toward GlcNAc was weakly correlated to total functional capacity, and stability of the native state and the levels of insoluble aggregates were not correlated (Figure 4C). Notably, the latter three factors were intercorrelated (Figure 4D; group 2). Indeed, the separation of group 1 and group 2 was primarily due to the identical fusion constructs (AncA_5B_ and Anc_5V_), which despite their total binding capacity being at mid range, showed the highest stability, binding affinity, and insoluble expression. This trend seems generalizable because AncA_5B_ is an exact 5-fold copy of the single motif AncA_1B_. Further, identical fusions showed similar behavior to one another, as did single motifs. The biophysical changes are therefore a direct result of tandem fusion irrespective of a precise amino acid composition. The mechanism underlining this correlation is discussed in later sections.

Beyond the correlation analysis, we also note that the total binding capacity of single motifs was 40-fold lower than WT (group 1), with 15- to 20-fold lower folding efficiency (group 1) and only 2- to 4-fold lower binding affinity (group 2). Indeed, when soluble, folded proteins were purified and assayed at identical propeller concentrations, most of the difference in their hemagglutination titer was lost (Table S1). Moreover, a relatively high binding affinity was already observed at the earliest evolutionary stage of the single motifs (Figure 4C), while the efficiency of folding evolved through the trajectory (Figure 4B). Overall, the above biophysical analysis indicates that despite inevitable uncertainties over the precise historical sequences, improved folding efficiency was the main parameter underlying the gradual increase in molecular fitness throughout the reconstructed trajectory.

### A Tradeoff between Pentamer Assembly and Intermolecular Misfolding of Single Motifs

**Figure 5:**
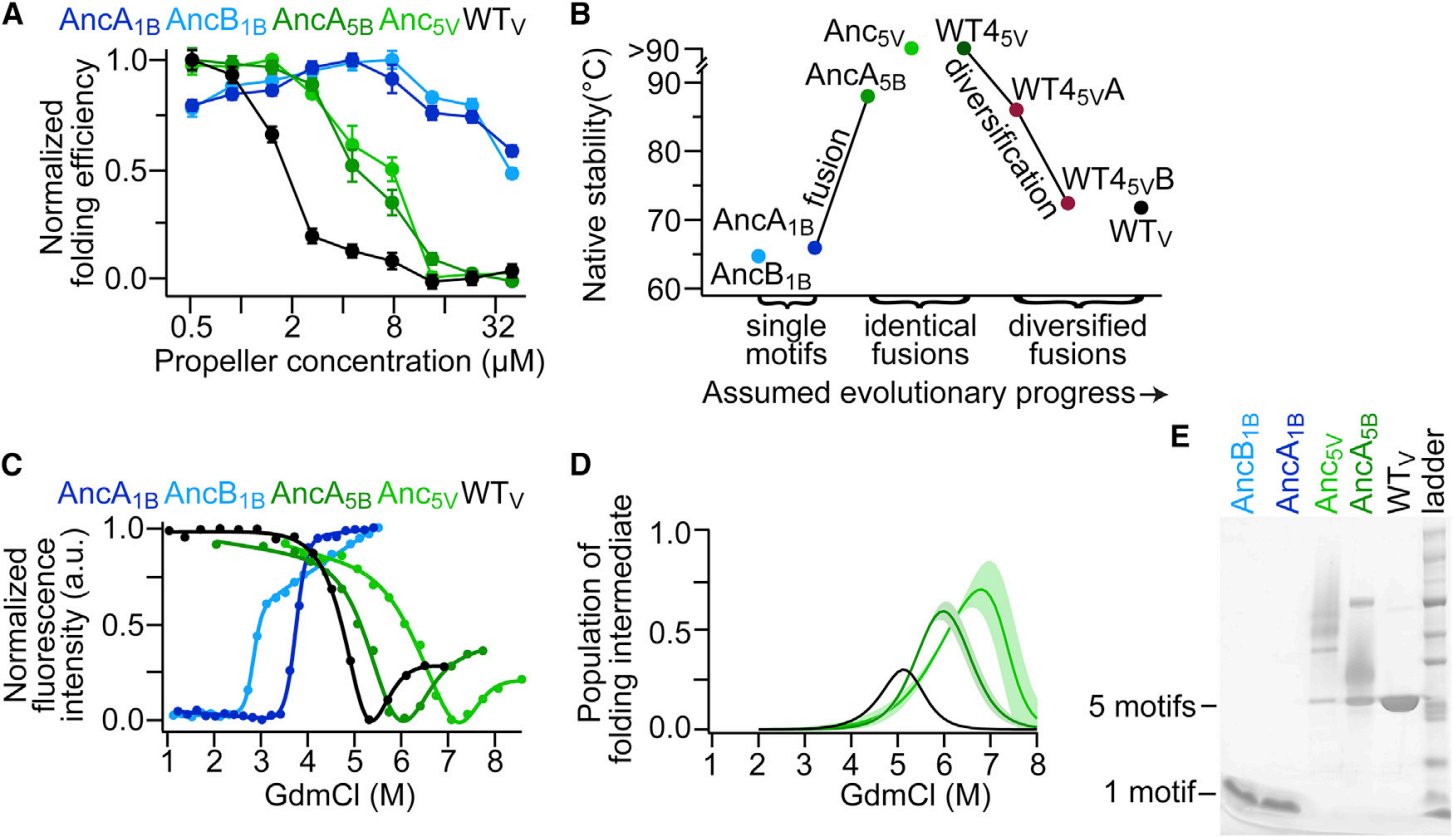
The Changing Roles of Intermolecular Assembly and Stability along the Evolutionary Trajectory (A) Intermolecular interactions were reshaped through the trajectory. Folding efficiency was measured at various concentrations and normalized to the maximum value for each construct (the absolute folding yields are shown in Figure 4B). Single motifs (blue) showed a bell-shaped concentration-dependence, thus indicating a tradeoff between native pentamerization and non-native intermolecular interaction leading to misfolding. In contrast, the monomeric tandem motif fusions folded most efficiently at low concentration where intermolecular interaction is minimized (green; WT in black). (B) When arranged by their evolutionary progression, a changing trend in native-state stability was observed: single-motif pentamers were moderately stable, monomeric identical fusions were hyperstable, and the selective diversification of identical fusions (WT4_5V_A and WT4_5V_B) returned to the moderate stability of WT_V_. Thermal unfolding was measured by CD. (C) Unfolding equilibria measurements at dilute protein concentration (0.5 μM) revealed a stable folding intermediate upon identical motif fusion. Pentameric single motifs were fit to a two-state folding model (blue), and the other constructs were fit to a three-state model (identical tandem fusions, green; WT, black). The stability associated with a folding intermediate between inflections (ΔC_m_) was 0.8 M GdmCl for AncA_5p_ and 1.2 M GdmCl for Anc_5_ versus 0.3 M GdmCl for WT. (D) The folding intermediates of identical tandem fusions were more populated than that of WT. The relative fractions of intermediates were extracted from the model fitting in (C), with SDs shown by shaded regions. (E) Identical tandem fusions formed misfolded, denaturation-resistant multimers more readily than WT. Natively folded propellers were incubated at high concentration (100 μM) and analyzed by SDS-PAGE. See also Figures S3 and S4 and Table S3.

As indicated above, foldability was the most dominant feature under selection. Poor foldability is often the result of non-native intermolecular between partially folded protein molecules, e.g., domain swaps, and is a known restrictive factor in β-rich proteins as well in repeat proteins (Borgia et al., 2011). The folding of monomers is typically optimal at low concentration where non-native intermolecular interactions are minimized. In contrast, at the onset of this trajectory, i.e., at the single motif, pentameric stage, the oligomeric assembly should be promoted at high concentration. The switch from an oligomer to a monomer is thus expected to affect intermolecular versus intramolecular folding demands. To examine these demands, the concentration dependence of folding efficiency was measured. The pentameric single motifs showed a bell-shaped curve of concentration-dependent folding efficiency, indicating that native pentamer formation was favored at higher concentrations but was simultaneously compromised by misfolding intermolecular interactions (Figure 5A; blue lines). In the tandem fusions, whereby the fundamental restriction of oligomer assembly was alleviated, native folding was strongly favored at low protein concentrations (Figure 5A; green lines). The consistent behavior of these constructs at each evolutionary stage indicated that, irrespective of a specific sequence context, the change from oligomer to monomer enhanced foldability, and thus, as indicated above (Figure 4), the increase in the levels of soluble, functional protein drove the evolutionary maturation.

### A Tradeoff between Stability and Foldability

The mechanistic origins of the correlation observed in Figure 4D were also examined. The correlation of native stability with binding affinity seems to relate primarily to the transition from the initial, oligomeric form to the duplicated-fused form, thereby leading to binding site stabilization. However, the correlation of native stability with insoluble expression was not immediately clear. When these data are viewed from the perspective of evolutionary progression, there is a consistent increase of native stability upon tandem fusion (Figure 5B). This increase relates to the entropic effect of fusion, as a 21°C increase in thermostability was observed with no change in sequence apart from fusion itself (AncA_1B_ to AncA_5B_). At the later evolutionary stage, the duplicated fusions (AncA_5B_, Anc_5V_, WT4_5V_) decreased in stability when selectively diversified (WT_V_). That higher foldability comes jointly with lower stability seemed counterintuitive—a stable native state suggests a deep native energy well, and thus smoother funneling and also lower tendency of the native state to misfold and aggregate, as routinely described for globular domains (Gillespie and Plaxco, 2000, Larson and Pande, 2003). Are the loss of native stability and the parallel decrease in levels of insoluble aggregates accompanying the transition from identical to diversified fusions a mechanistic underpinning of higher foldability, or are they perhaps the result of selection for another biophysical property or simply the outcome of most mutations having a destabilizing effect (Tokuriki et al., 2007)?

We first examined the robustness of the above trend by examining the identical tandem fusion of WT fourth sequence repeat (WT4_5V_) and its selected diversification pathway to WT4_5V_A and WT4_5V_B. As observed with the ancestral fusions, the selected mutations leading to higher foldability also resulted in a loss of stability (Figure 5B) well beyond the expected change for few typically destabilizing mutations (Tokuriki et al., 2007). The mechanistic basis underlying this trend was revealed by closer examination of the folding pathways along the evolutionary trajectory. Unfolding equilibria were measured by monitoring tryptophan fluorescence as a function of denaturant concentration. As above, the various evolutionary stages were represented by the oligomeric single motifs AncA_1B_ and AncB_1B_, the identical fusion monomers AncA_5B_ and Anc_5V_, and the diversified WT monomer. Single motifs unfolded in a simple two-state transition (Figure 5C). However, a three-state transition with a stable folding intermediate appeared in the identical fusions and persisted in WT, underscoring a substantial alteration of the folding landscape upon motif fusion.

**Figure S4:**
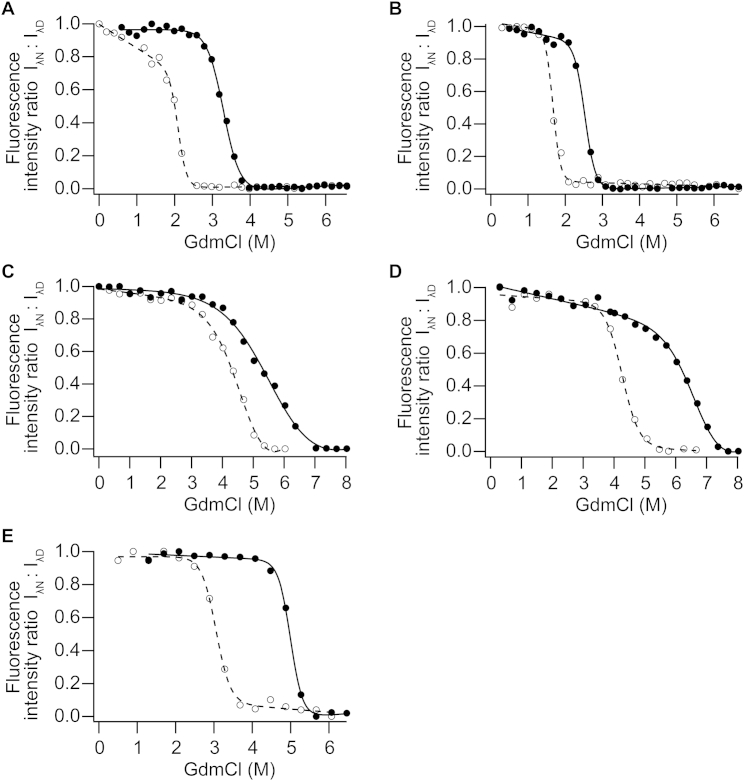
Unfolding Parameters by Two-State Fitting, Related to Figure 5 Unfolding equilibria of constructs were again measured by incubating purified protein in GdmCl and measuring fluorescence spectra, as before (Figure 5C). However, in order to determine any dependence on the parameterization of curve fitting, the data in this case were first transformed to two states. At each denaturant concentration, fluorescence intensities at wavelengths corresponding to native and denatured states were taken as a ratio (I_λN_:I_λD_). Two-state unfolding models were fit for single motifs (A) AncA_1B_ and (B) AncB_1B_, identically fused constructs (C) AncA_5B_ and (D) Anc_5V_ and (E) WT_V_, in the presence (filled circles) and absence (empty circles) of GlcNAc. Intermediate populations observed for identical fusions and WT (Figures 5C and 5D) manifest here as a less cooperative native-denatured unfolding transition with milder slope (lower pseudo-*m* value; Table S3).

The native stability determined using a chemical denaturant (C_m_), i.e., the first inflection in unfolding, followed the same trend as that determined by thermal melts (Figures 5B and 5C; Table S3): namely, moderately stable single motifs, hyperstabilization upon identical fusion, and a return to moderate stability upon diversification to WT sequence. The unfolding intermediates of tandem fusions were also highly stable with a broad interval between inflections (ΔC_m_) (Figure 5C; Table S3). Accordingly, the unfolding intermediate was more highly populated for tandem fusions than for the single motifs and WT (Figure 5D). These differences among constructs were also supported by independent datasets measured in the presence and absence of sugar ligand (GlcNAc) and analyzed using the parsimonious option of a two-state transformation to assess any dependency on curve fitting (Figure S4; Table S3).

A populated folding intermediate is associated with proclivity for aggregation (Neudecker et al., 2012). This suggests a mechanistic link between hyperstability of the native state of the identical tandem fusions and their high aggregation propensity, with the latter being due to the accumulation of a highly stable folding intermediate. This coupling seems inevitable because the very same interactions that stabilize the native assembly of tandem fusions can equivalently stabilize domain-swapped misfolded forms (Borgia et al., 2011, Zheng et al., 2013). Vulnerability to aggregation under mild, non-denaturing buffer conditions was also tested by prolonged incubation of natively folded and functional lectins at high concentration and subjecting them to brief denaturation in SDS-PAGE (Figure 5E). In further support of the proposed mechanistic link, single motifs were found exclusively in a disassociated form, whereas tandem fusions showed extensive formation of midfolded, denaturation-resistant multimers. The latter were reduced and largely disappeared upon diversification. Thus, the demands on foldability changed during the evolutionary trajectory. A moderately stable oligomeric assembly enabled the emergence of functional single motifs. Upon their fusion, the native state became hyperstable due to the large entropic change associated with fusion, but this also led to the stabilization of an aggregation-prone folding intermediate. Improvements in foldability demanded the destabilization of this intermediate. However, because of the high coupling between the stability of this intermediate and that of the final, native state, improved foldability traded off with native stability.

### Genomic Evidence of Propeller Origins

We established the functionality of single ancestral motifs that relate to tachylectin-2’s contemporary sequence. We did not, however, observe a single tachylectin motif in extant genomes, likely because ancestral states are relatively short lived. Nonetheless, as described earlier, nearly 1,000 extant propellers show 50%–100% internal sequence identity, thus supporting a mechanism of emergence by motif duplication and fusion. These weakly diverged sequences were next used as query to systematically search for related single motif sequences within the same genomes.

**Figure 6:**
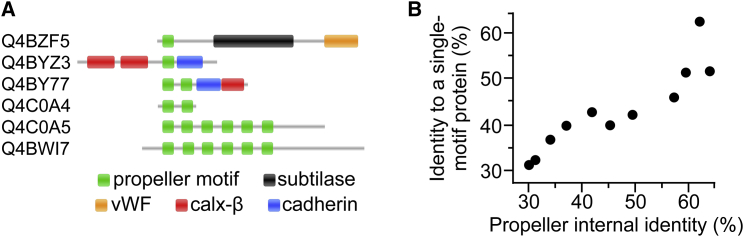
Single-Motif Proteins Are Found in Genomes and Follow a Mechanism of Duplication, Fusion, and Divergence (A) Single motifs (42 amino acids, one green bar) were identified in *C. watsonii* open reading frames with 37%–65% sequence identity to six-motif β-propellers proteins (average identity for all propeller motifs) within the same genome. Proteins are labeled with their Uniprot accession codes. (B) Molecular clock divergence posits that “young” propellers that are less diverged from ancestral states should closely resemble an originating single motif and preserve high internal sequence identity. In a *Frankia sp*. strain, this correlation was observed among a large collection of five- to seven-motif propellers and a single motif protein without non-propeller domains (Pearson’s r = 0.91, p < 0.001). See also Figures S5 and S6, Table S6, and Data S1.

**Figure S5:**
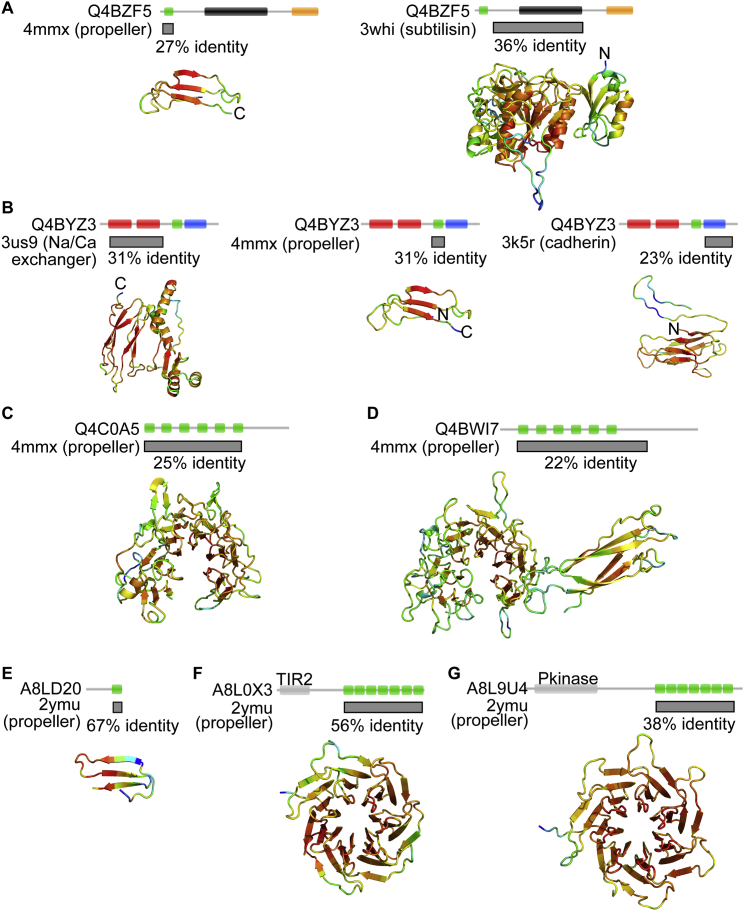
Homology-Modeled Structural Domains of Genomic Sequences, Related to Figure 6 Sequences containing propeller motifs from (A–D) *C. watsonii* and (E–G) *Frankia* sp. strain EAN1pec were modeled with SwissProt using homologs from the pdb. For each panel, the overlap with the pdb sequence is shown by a gray box with the corresponding homology model shown below. (A and B) Single-propeller motifs are flanked by domains that do not form propellers, and accordingly, the homology model indicates non-propeller folds. One *C. watsonii* gene is addressed per row. The homology model for the single motif (denoted in green in the sequence diagram and typically modeled as four-stranded β sheet) is shown, alongside the predicted models for the flanking domains. (C and D) Genes composed of six propeller motifs are predicted to form six of the seven blades of a distant propeller homolog, suggesting that they close the radially arranged blades to form intact propellers. (E) An identified *Frankia* gene containing a single propeller motif. (F and G) Genes encoding multi-motif propellers. For all panels, Qmean4 scores (Benkert et al., 2009) given by Swiss-model (Biasini et al., 2014) indicate physical features of model quality and indicate that the predicted structures are most reliable in core structural regions, as shown by residue coloring using a Qmean4 heat map (blue = low to red = high). Uniprot accession codes are labeled.

The search discovered ancestral single motifs, curiously in bacterial genomes, although propellers are most prevalent in eukaryotes (Figure 6). For example, the cyanobacterium *C. watsonii* contains a series of homologous sequences containing one, two, and six propeller motifs, showing 63%–76% identity between the repeating motifs of the same protein and 37%–65% identity to a single motif within another protein (Figure 6A). The single motifs are flanked by domains belonging to non-propeller folds, suggesting that a β sheet motif with a propeller-forming potential was co-opted to generate a propeller (Figures S5A–S5D). While functionally uncharacterized, the cyanobacterial propellers show sequence homology to FG-GAP β-propeller motifs (found in integrin α), vWF (found in integrin α and β), calxβ (found in integrin β4), and cadherin (Figure 6A), suggesting roles in cell signaling and adhesion.

**Figure S6:**
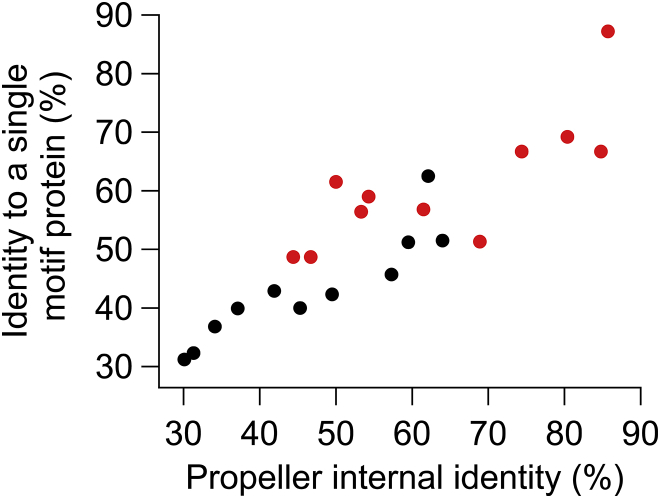
Directionality of Emergence in *Frankia* Propeller Motifs, Related to Figure 6 In a scenario where a single motif was duplicated, fused, and gave a propeller, all propeller repeats would be equally diverged with respect to the single motif and with respect to one another. In the reverse scenario, however, in which a single motif from an existing propeller was duplicated and inserted into another protein, the propeller repeats would be equally diverged with respect to one another, but the single motif would resemble one repeat more than the others. To test this idea, average identities (black dots, as in Figure 6B) were compared against maximum identities (red dots) in a molecular clock plot. Overall, in agreement with the first scenario, the maximum and average identities hold the same trends, indicating that the single motif does not disproportionately resemble one repeat more than the others.

More evidence for the de novo emergence of propellers was found in homologous single motifs from 20 bacterial species, with the greatest representation in cyanobacteria such as *C. watsonii* and actinobacteria such as *Frankia* sp. strain EAN1pec (Data S1). These proteins are typically short (median length of 81 residues) with a single propeller-like motif. The *Frankia* genome in particular contains a single motif protein and a large number of homologous repeat proteins predicted as propellers (32%–56% identity to a known WD40 propeller, PDB 2YMU; Figures S5E–S5G). These 11 propellers each comprise five to seven tandem motifs with a wide range of average internal identity (30%–62%) and thus allowed meaningful statistics to test the relationship of common ancestry by molecular clock divergence within the very same genome (Figure 6B). Following the model of motif duplication, fusion, and diversification, the age of propellers relates to their level of internal sequence identity, with the “youngest” propellers showing the highest internal identity. The molecular clock analysis supports this model of emergence. The internal motifs of the younger propellers were the least diverged from their putative single motif ancestors, and this correlation persisted through a wide range of divergence (Figure 6B). The reverse scenario may also apply, i.e., co-option of a single motif from an existing propeller. However, this seems unlikely because only transfer and co-option from a propeller with high internal identity (<1% of total propellers) would produce the trend seen in Figure 6B. Moreover, the single motifs did not disproportionately resemble any of the individual propeller motifs, as expected by this alternative scenario (Figure S6).

Sequences composed of two or three non-identical repeats were also found (in addition to one sequence with four fully identical motifs). These support routes that are complementary to the basic trajectory explored here (Figure 2), including intermediates of partial duplication that considerably diversify prior to the final duplication step. An alternative route suggested by the genomic data is that co-opted single propeller motif may be initially functional with additional sequence elements, rather than the bare single motifs explored here. Overall, the above genomic analysis and the bacterial context in particular indicate that propellers readily emerge de novo and via multiple parallel routes.

## Discussion

Single motifs are considered foundational to the emergence of symmetrical proteins (Balaji, 2015). We observed that single motifs (<50 amino acids) reconstructed from contemporary propeller sequences have biochemical activity (Figure 3A), and such motifs also appear to exist in bacterial genomes (Figure 6). Overall, we found that propeller emergence is a likely event, in the sense that several alternative complementary pathways may lead to folded and functional propellers (Figure 2). In experiments, we examined the maturation pathway of motif expansion followed by internal diversification, and these trajectories seem to be compatible with little predetermined order. For example, hundreds of single motifs with detectable function were isolated from ancestral libraries, or the F23L mutation appeared upon selection of single motifs and also upon selection of the fused motifs. Further, beyond the single oligomerizing motif, the frame was flexible, with repeat proteins tolerating the ancestral blade frame as well as the WT Velcro frame. The transition state between these two frames, namely six-repeat proteins, is also viable (Figure 3).

Evolutionary trajectories that are parallel and/or complementary to the most basic trajectory followed here (Figure 2) include intermediate tandem fusions of two to three motifs that already begin to diversify and the inclusion of additional domains fused to single motifs (Figure 6A). The evolutionary feasibility of the former is also supported by two-repeat fragments of tachylectin-2 being functional (Yadid et al., 2010). These alternative routes are expected to affect the selection properties beyond the basic features described here.

The main parameter shaping the total binding capacity, or “fitness” at the protein level, was folding efficiency, or foldability. Expansion of the single, oligomerizing motifs to fully symmetrical proteins (five identical motifs) alleviated the folding constraints imposed by linked concentration-dependent folding and misfolding observed for oligomers (Figure 5A). Native stability also increased substantially as a direct entropic consequence of motif fusion, but this led to the parallel stabilization of an intermediate that seems to be associated with misfolding and aggregation (Figure 5).

Indeed, another finding of this work regards the seemingly paradoxical tradeoff between stability and foldability in symmetrical proteins. Domain-swapped misfolding is a common property of proteins with high internal sequence identity. Repeating sequence elements can form the same interactions in natively folded and alternatively misfolded topologies, resulting in a rugged, frustrated folding process (Borgia et al., 2011, Zheng et al., 2013), as observed in the emergence of a stable, aggregation-prone folding intermediates of identical fusions (Figures 5C and 5D). Accordingly, the same interactions that stabilize the native, intramolecular assembly of tandem fusions are also likely to stabilize domain-swapped misfolded forms and intermolecular interaction leading to aggregation. Given the near identity of these competing forms, selective diversification sacrificed the stability of all, and specificity of interactions came at the expense of stability (Lumb and Kim, 1995). Thus, high native-state stability and efficient folding were not correlated as usually observed. In contrast, they became anti-correlated upon tandem fusion of identical motifs (Figure 5B). Overall, protein stability is a complex property that also influences the binding site and thereby the binding affinity of these propeller domains (Figure 4D). Selection therefore shapes in parallel several different traits that relate to stability, including folding smoothness and folding and unfolding rates (Broom et al., 2015). This work shows how some of these traits can trade off and shape the evolutionary trajectories that lead to new proteins.

## Experimental Procedures

### Bioinformatics

Propeller sequences were collected from Pfam clan CL0186 (226,440 sequences, 95,306 non-redundant; circa 2013). Internal sequence repeats were detected and aligned using Radar (Heger and Holm, 2000). The parent genomes of Pfam sequences with ≥50% internal identity were collected from the EMBL-EBI database using dbFetch. Single-motif proteins within the same genome of a Pfam query were searched by homology (Blast e-value < 10^−3^) and number of motifs (Radar).

### Ancestral Inference and Libraries

*T. tridentatus* and *N. vectensis* tachylectin-2 motifs were split and aligned for ancestral reconstruction (Figure S1). Ancestral motif reconstructions were made with maximum likelihood prediction in FastML (Ashkenazy et al., 2012) by taking the posterior probabilities at the root node of their phylogenetic tree (Figure S1). Alternatively, the same analysis was performed with *T. tridentatus* alone, and natural substitutions were chosen that exceeded probability cutoffs by either analysis (>0.25 in the first round and >0.1 in the second round). The consensus sequence motif of WT tachylectin-2 (the fourth repeat) was identified by the highest average identity to other motifs of *T. tridentatus and N. vectensis*. Motif libraries were constructed by overlap extension PCR of synthetic oligonucleotides to generate a megaprimer that was extended by ligation-free cloning into pET29. The alternatively predicted amino acids contained by oligonucleotides and their combinatorial inclusion in complete motifs by overlap extension are detailed in Supplemental Experimental Procedures. Random mutagenesis, resulting in AncB_1B_, WT4_5V_A, and WT4_5V_B, was performed by error-prone PCR using Mutazyme II DNA polymerase (Stratagene). Tandem fusions of identical motifs were made by iterative motif ligation using type IIS restriction sites as described (Yadid and Tawfik, 2011) or by gene synthesis (Genscript). See Table S4 for the sequences of constructs.

### ELISA and Protein Partitioning

ELISA was performed using cell lysates or purified proteins as described (Yadid et al., 2010). Briefly, mucin (porcine stomach type II; Sigma) was coated on multiwell ELISA plates, and lectin binding was detected by the subsequent binding of rabbit polyclonal anti-tachylectin-2 serum followed by an anti-rabbit HRP-linked antibody (Sigma) and TMB+ as substrate (Dako). Following initial screening, plasmids were isolated from selected clone and retransformed, and ELISA was remeasured in three independent biological replicates. Background absorbance was subtracted using cells transformed with empty plasmid. The reported total binding capacity was determined from cell lysate samples corresponding to 0.8 μg pre-lysis dry weight, a quantity that gave a reliable signal within the dynamic range of ELISA for all constructs. The total binding capacity was measured as the raw ELISA absorbance of lysate, calibrated from the non-linear scaling of ELISA to levels of protein function (Figure S2A) and normalized for batch variation in each run with a reference WT lysate ELISA. This approach gave quantitatively reproducible comparisons between constructs that also correlated with hemagglutination titers (Table S1). Partitioning of expressed variants into soluble and insoluble fractions was estimated as described (Tomoyasu et al., 2001) using the same lysates assayed by ELISA. Coomassie-stained gels were scanned and bands of interest were quantified by peak analysis with baseline subtraction using ImageJ.

### Folding Efficiency and Binding Affinity

Proteins were purified as previously described with GlcNAc-linked agarose resin (Sigma) and GlcNAc elution (Yadid and Tawfik, 2011). For in vitro refolding, samples were denatured in 8 M guanidinium chloride (GdmCl), renatured by 10-fold dilution into 20 mM Tris, 150 mM NaCl (pH 7.6), and the renaturation yield was monitored by activity in ELISA. Non-denatured samples kept in renaturation buffer served as control. Alternatively, thermal denaturation was performed by heating to 99°C for 30 min, refolding by cooling 1°C per minute to 25°C, and determining the natively refolded fraction by ELISA. Refolding efficiency was determined by the fractional refolded:control ELISA signal. Isothermal titration calorimetry (Microcal ITC200) was applied with 10 μM propeller variants at 25°C in 20 mM Tris, 150 mM NaCl (pH 7.6) with serial injection of GlcNAc. The data fit best to a single site binding model, suggesting nearly identical and non-cooperative binding sites.

### Size-Exclusion Chromatography

Proteins at 0.8–12 μM propeller concentrations were run on a GE HiLoad 26/60 Superdex 75 column and were eluted with 20 mM Tris, 150 mM NaCl (pH 7.6).

### X-Ray Crystallography

See Supplemental Experimental Procedures and Table S5.

### CD

The CD spectra of propeller variants were recorded in 20 mM sodium phosphate buffer (pH 7.6) (Applied Photophysics). For temperature melts, ellipticity was recorded at 202 nm with a heating rate of 1°C per minute. In cases of incompletely determined denaturation baselines, the dependency of the normalized denaturation determined by CD was validated by residual activity measurement using ELISA, and in some cases, the unfolding midpoint was qualitatively limited to >90°C.

### Folding Equilibria

A 0.5 μM propeller was incubated in a buffered GdmCl concentration gradient for 1–2 days with 20 mM Tris, 150 mM NaCl, 10 mM GlcNAc (pH 7.6), followed by measurement of tryptophan fluorescence (Varian Cary Eclipse). Maximal fluorescence intensities from each spectrum were plotted as a function of GdmCl concentration and fit to either two- or to three-state folding models, as appropriate (Santoro and Bolen, 1988). Propellers were kept at dilute concentration (0.5 μM) and in the presence of GlcNAc to improve refolding yields, but unfolding/refolding cycles were still not fully reversible and thus the fit parameters were considered as apparent values (see also Supplemental Experimental Procedures).

### Aggregation

Natively folded proteins gave single bands of monomers in SDS-PAGE. To visualize formation of denaturation-resistant multimers, 100 μM native propellers were stored for 2 months at 4°C in 20 mM Tris, 150 mM NaCl (pH 7.6) and analyzed. Samples were resolved on SDS-PAGE upon incubation in SDS gel-loading buffer for 10 min at room temperature.

## Author Contributions

R.G.S., I.Y., J.C., and D.S.T. designed experiments. R.G.S. performed experiments, except characterization of WT4_5_ constructs and *N. vectensis* tachylectin-2 were performed by I.Y. and x-ray crystallography was performed by O.D. R.G.S. and D.S.T. wrote the manuscript.

## Accession Numbers

The accession numbers for the coordinates of AncB_1B_ and *N. vectensis* tachylectin-2 reported in this paper are RCSB Protein Data Bank: 5C2N and 52CM, respectively.

## Supplemental Information

Document S1. Supplemental Experimental Procedures and Tables S1–S6

Data S1. Propeller Motifs in *C. Watsonii* and *Frankia* sp. Strain EAN1pec, Related to Figure 6 In *C. watsonii*, two single-motif proteins (Uniprot: Q4BZF5 and Q4BYZ3) each contain 41–42 amino acids that closely correspond to the FG-GAP propeller motif described in Pfam. In the other multimotif proteins, the core motifs are also extended with sequence that preserves continuous tandem repetition. In *Frankia*, there is one single-motif protein (Uniprot: A8LD20) homologous to the tandemly repeating motifs of many structural propellers of the same organism and to the single motif proteins of other organisms.

Document S2. Article plus Supplemental Information

## References

[bib1] Afriat-Jurnou L., Jackson C.J., Tawfik D.S. (2012). Reconstructing a missing link in the evolution of a recently diverged phosphotriesterase by active-site loop remodeling. Biochemistry.

[bib2] Ashkenazy H., Penn O., Doron-Faigenboim A., Cohen O., Cannarozzi G., Zomer O., Pupko T. (2012). FastML: a web server for probabilistic reconstruction of ancestral sequences. Nucleic Acids Res..

[bib3] Balaji S. (2015). Internal symmetry in protein structures: prevalence, functional relevance and evolution. Curr. Opin. Struct. Biol..

[bib4] Bar-Rogovsky H., Stern A., Penn O., Kobl I., Pupko T., Tawfik D.S. (2015). Assessing the prediction fidelity of ancestral reconstruction by a library approach. Protein Eng. Des. Sel..

[bib5] Bershtein S., Mu W., Serohijos A.W., Zhou J., Shakhnovich E.I. (2013). Protein quality control acts on folding intermediates to shape the effects of mutations on organismal fitness. Mol. Cell.

[bib6] Borgia M.B., Borgia A., Best R.B., Steward A., Nettels D., Wunderlich B., Schuler B., Clarke J. (2011). Single-molecule fluorescence reveals sequence-specific misfolding in multidomain proteins. Nature.

[bib7] Bridgham J.T., Ortlund E.A., Thornton J.W. (2009). An epistatic ratchet constrains the direction of glucocorticoid receptor evolution. Nature.

[bib8] Broom A., Doxey A.C., Lobsanov Y.D., Berthin L.G., Rose D.R., Howell P.L., McConkey B.J., Meiering E.M. (2012). Modular evolution and the origins of symmetry: reconstruction of a three-fold symmetric globular protein. Structure.

[bib9] Broom A., Gosavi S., Meiering E.M. (2015). Protein unfolding rates correlate as strongly as folding rates with native structure. Protein Sci..

[bib10] Chen S., Krinsky B.H., Long M. (2013). New genes as drivers of phenotypic evolution. Nat. Rev. Genet..

[bib11] Claren J., Malisi C., Höcker B., Sterner R. (2009). Establishing wild-type levels of catalytic activity on natural and artificial (β α)8-barrel protein scaffolds. Proc. Natl. Acad. Sci. USA.

[bib12] Gillespie B., Plaxco K.W. (2000). Nonglassy kinetics in the folding of a simple single-domain protein. Proc. Natl. Acad. Sci. USA.

[bib13] Heger A., Holm L. (2000). Rapid automatic detection and alignment of repeats in protein sequences. Proteins.

[bib14] Hingorani K.S., Gierasch L.M. (2014). Comparing protein folding in vitro and in vivo: foldability meets the fitness challenge. Curr. Opin. Struct. Biol..

[bib15] Höcker B. (2014). Design of proteins from smaller fragments-learning from evolution. Curr. Opin. Struct. Biol..

[bib16] Kopec K.O., Lupas A.N. (2014). β-propeller blades as ancestral peptides in protein evolution. PLoS ONE.

[bib17] Larson S.M., Pande V.S. (2003). Sequence optimization for native state stability determines the evolution and folding kinetics of a small protein. J. Mol. Biol..

[bib18] Lee J., Blaber M. (2011). Experimental support for the evolution of symmetric protein architecture from a simple peptide motif. Proc. Natl. Acad. Sci. USA.

[bib19] Longo L.M., Blaber M. (2014). Symmetric protein architecture in protein design: top-down symmetric deconstruction. Methods Mol. Biol..

[bib20] Longo L.M., Lee J., Tenorio C.A., Blaber M. (2013). Alternative folding nuclei definitions facilitate the evolution of a symmetric protein fold from a smaller peptide motif. Structure.

[bib21] Lumb K.J., Kim P.S. (1995). A buried polar interaction imparts structural uniqueness in a designed heterodimeric coiled coil. Biochemistry.

[bib22] Merkl R., Sterner R. (2015). Ancestral protein reconstruction: techniques and applications. Biol. Chem..

[bib23] Nepomnyachiy S., Ben-Tal N., Kolodny R. (2014). Global view of the protein universe. Proc. Natl. Acad. Sci. USA.

[bib24] Neudecker P., Robustelli P., Cavalli A., Walsh P., Lundström P., Zarrine-Afsar A., Sharpe S., Vendruscolo M., Kay L.E. (2012). Structure of an intermediate state in protein folding and aggregation. Science.

[bib25] Park K., Shen B.W., Parmeggiani F., Huang P.-S., Stoddard B.L., Baker D. (2015). Control of repeat-protein curvature by computational protein design. Nat. Struct. Mol. Biol..

[bib26] Peisajovich S.G., Rockah L., Tawfik D.S. (2006). Evolution of new protein topologies through multistep gene rearrangements. Nat. Genet..

[bib27] Santoro M.M., Bolen D.W. (1988). Unfolding free energy changes determined by the linear extrapolation method. 1. Unfolding of phenylmethanesulfonyl α-chymotrypsin using different denaturants. Biochemistry.

[bib28] Tokuriki N., Stricher F., Schymkowitz J., Serrano L., Tawfik D.S. (2007). The stability effects of protein mutations appear to be universally distributed. J. Mol. Biol..

[bib29] Tomoyasu T., Mogk A., Langen H., Goloubinoff P., Bukau B. (2001). Genetic dissection of the roles of chaperones and proteases in protein folding and degradation in the Escherichia coli cytosol. Mol. Microbiol..

[bib30] Vellieux F.M., Huitema F., Groendijk H., Kalk K.H., Jzn J.F., Jongejan J.A., Duine J.A., Petratos K., Drenth J., Hol W.G. (1989). Structure of quinoprotein methylamine dehydrogenase at 2.25 A resolution. EMBO J..

[bib31] Verstrepen K.J., Jansen A., Lewitter F., Fink G.R. (2005). Intragenic tandem repeats generate functional variability. Nat. Genet..

[bib32] Voet A.R., Noguchi H., Addy C., Simoncini D., Terada D., Unzai S., Park S.Y., Zhang K.Y., Tame J.R. (2014). Computational design of a self-assembling symmetrical β-propeller protein. Proc. Natl. Acad. Sci. USA.

[bib33] Wright C.F., Teichmann S.A., Clarke J., Dobson C.M. (2005). The importance of sequence diversity in the aggregation and evolution of proteins. Nature.

[bib34] Yadid I., Tawfik D.S. (2007). Reconstruction of functional β-propeller lectins via homo-oligomeric assembly of shorter fragments. J. Mol. Biol..

[bib35] Yadid I., Tawfik D.S. (2011). Functional β-propeller lectins by tandem duplications of repetitive units. Protein Eng. Des. Sel..

[bib36] Yadid I., Kirshenbaum N., Sharon M., Dym O., Tawfik D.S. (2010). Metamorphic proteins mediate evolutionary transitions of structure. Proc. Natl. Acad. Sci. USA.

[bib37] Zheng W., Schafer N.P., Wolynes P.G. (2013). Frustration in the energy landscapes of multidomain protein misfolding. Proc. Natl. Acad. Sci. USA.

