# Supplementary material for: De Novo Evolutionary Emergence of a Symmetrical Protein Is Shaped by Folding Constraints
Source: Cell. 2016 Jan 28;164(3):476–86. doi: 10.1016/j.cell.2015.12.024 (PMC4735018; doi:10.1016/j.cell.2015.12.024)
Supplement: Document S1. Supplemental Experimental Procedures and Tables S1–S6 [file mmc1.pdf]

Cell

Supplemental Information

De Novo Evolutionary Emergence of a Symmetrical Protein Is Shaped by Folding Constraints

Robert G. Smock, Itamar Yadid, Orly Dym, Jane Clarke, and Dan S. Tawfik

SUPPLEMENTAL EXPERIMENTAL PROCEDURES

Ancestral Permutation and Substitution

In first round libraries of single motifs, sequences were constructed in the wildtype Velcro frame (such as Anc_{IV}) and were also topologically permuted to five additional frames corresponding to an intact structural blade that removed Velcro closure (such as Anc_{1B}). Permuted frames corresponded to wildtype motif positions (41-47,1-40) ±2 residues at the termini (see **Figure S1B**). Motif libraries were constructed with ancestral substitutions that were predicted with ancestral probability >0.25, resulting in ~10^3 unique sequences per permutation frame. Given the reference sequence: MNNFKFLFFSPNGTLYGVHGDKFYKGTPPQNDNDNWLARATKIGNGGW, the following combinatorial substitutions were made in the first round library: 2NS, 3GN, 5KR, 10NS, 12DGNS, 20GN, 30QT, 31HNS, 41KT, and 42KL. Equivalent substitutions were made in each of the six permutation frames. However, functional motifs were observed only in a frame corresponding to wildtype motif positions (40-47,1-39) (**Figure S1B**). A second round library was built from the most functional sequence of the first round library (given in **Table S4**). This library was expanded to ~10^4 sequences by fixating convergent mutations from the first round and including other ancestral substitutions predicted with ancestral probability >0.1: 2DGNS, 3DGNQ, 14MT, 19HLQ, 20GKN, 27ST, 30QST, 31HNSY, 37LM, and 41KT.

Unfolding Equilibria (Two-State Comparison)

Raw fluorescence spectra of unfolding equilibria were reproduced, as described in the main text. In **Figure 5C**, the maximum fluorescence intensity at any wavelength was plotted. Here, spectra were analyzed in a parametrically reduced two-state transformation. For each fluorescence spectrum at a given GdmCl concentration, the fluorescence intensities at wavelengths corresponding to the native and denatured states were taken as a ratio and plotted. The ratio made an internal correction for erroneous intensity fluctuations between measurements and also allowed comparison of all constructs in a two-state transformation with less fitting parameters. Data were fit to a two-state folding model (Santoro and Bolen, 1988).

Structural Determination and Refinement of $AncB_{1B}$

Following expression and purification using GlcNAc-agarose resin and gel filtration, $AncB_{1B}$ was concentrated to 17 mg/ml in buffer composed of 20 mM Tris, 50 mM NaCl, pH 7.6. GlcNAc was added at a 1:1 molar ratio with protein. Crystals of $AncB_{1B}$ were obtained using the sitting drop vapor diffusion method with a Mosquito robot (TTP LabTech). The crystals were grown from 0.05 M PCB buffer (2:1:2 ratio of sodium propionate, sodium cacodylate and Bis-Tris propane, pH 7) and 12% polyethylene glycol 1,500. The crystals formed in the trigonal space group $P3_112$, with cell constants a=b=112.22, and c=127.50 Å and γ=120°. $AncB_{1B}$ crystallized with 15 monomers per asymmetric unit comprising three pentamers. A complete dataset to 1.65 Å resolution was collected at 100 K on a single crystal at an in-house Rigaku RAxis IV++ generator with an RU-H3R rotating anode generator. Diffraction images were indexed and integrated using the Mosflm program (Leslie and Powell, 2007), and the integrated reflections were scaled using the SCALA program (Evans, 2006). Structure factor amplitudes were calculated using TRUNCATE (French and Wilson, 1978) from the CCP4 program suite. The structure was solved by molecular replacement with the program PHASER (McCoy, 2007) using the refined structure of the evolved pentameric lectin (PDB: 3KIH) as a model. All steps of atomic refinements were carried out with the CCP4/REFMAC5 program (Murshudov et al., 1997). The model was built into $2mF_{obs} - DF_{calc}$, and $mF_{obs} - DF_{calc}$ maps by using the COOT program (Emsley and Cowtan, 2004). At early stages of refinement, non-crystallographic symmetry was restrained, and at later stages it was gradually released, followed by concomitant decrease in R_{free}. Refinement movements were accepted only when they produced a decrease in the R_{free} value. The $AncB_{1B}$ construct is composed of 48 amino acid residues and the final model includes residues 2-48 for 10 monomers and 3-48 for 5 monomers, 500 water molecules and one GlcNAc ligand per monomer. The R_{free} value is 27.7% (for the 5% of reflections not used in the refinement), and the R_{work} value is 18.6 % for all data to 1.65 Å. The $AncB_{1B}$ model was evaluated with the PROCHECK program (Laskowski et al., 1993) and validated with MolProbity (Chen et al., 2010). Details of the refinement statistics are described in **Table S5**.

Structural Determination and Refinement of *N. vectensis* Tachylectin-2

N. vectensis tachylectin-2 was cloned from cDNA into a pET20b vector with its leader peptide replaced by a His_6 tag. Following expression in *E. coli* BL21(DE3), the protein was purified in two steps using Ni-NTA resin and gel filtration. The purified protein was dialyzed in PBS and concentrated to 13 mg/ml. Crystals were obtained under oil by the microbatch method and an Oryx6 robot (Douglas Instruments). The crystals were grown from 0.1M NaF, 0.05 M Bis-Tris propane, pH 7.5, and 10% polyethylene glycol 3,350. Crystals formed in the orthorhombic space group $P2_12_12_1$, with cell constants a=44.69, b=60.93, and c=88.35 Å. Protein crystallized with one monomer per asymmetric unit. A complete dataset to 1.9 Å resolution was collected at 100 K on a single crystal on an in-house x-ray source. Diffraction images were indexed and integrated using HKL2000 (Otwinowski and Minor, 1997). Integrated reflections were scaled using the program SCALEPAC. Structure factor amplitudes were calculated using TRUNCATE from the CCP4 program suite. The structure was solved by molecular replacement with the program PHASER, using the refined structure of *T. Tridentatus* tachylectin-2 (PDB: 1TL2) as a model. The *N. vectensis* tachylectin-2 construct is composed of 249 amino acid residues and the final model includes residues 13-249. The R_{free} value is 23.5% (for the 5% of reflections not used in the refinement), and the R_{work} value is 19.1% for all data to 1.9 Å. The model was evaluated with the PROCHECK program and validated with MolProbity (Chen et al., 2010). Details of the structural refinement statistics are described in **Table S5**.

SUPPLEMENTAL REFERENCES

Benkert, P., Kunzli, M., and Schwede, T. (2009). QMEAN server for protein model quality estimation. Nucleic Acids Res *37*, W510-514.

Biasini, M., Bienert, S., Waterhouse, A., Arnold, K., Studer, G., Schmidt, T., Kiefer, F., Cassarino, T.G., Bertoni, M., Bordoli, L., *et al.* (2014). SWISS-MODEL: modelling protein tertiary and quaternary structure using evolutionary information. Nucleic Acids Res *42*, 252-258.

Chen, V.B., Arendall, W.B., 3rd, Headd, J.J., Keedy, D.A., Immormino, R.M., Kapral, G.J., Murray, L.W., Richardson, J.S., and Richardson, D.C. (2010). MolProbity: all-atom structure validation for macromolecular crystallography. Acta Crystallogr D Biol Crystallogr *66*, 12-21.

Emsley, P., and Cowtan, K. (2004). Coot: model-building tools for molecular graphics. Acta Crystallogr D Biol Crystallogr *60*, 2126-2132.

Evans, P. (2006). Scaling and assessment of data quality. Acta Crystallogr D Biol Crystallogr *62*, 72-82.

French, S., and Wilson, K. (1978). On the treatment of negative intensity observations. Acta Crystallogr *34*, 517-525.

Laskowski, R.A., MacArthur, M.W., Moss, D.S., and Thornton, J.M. (1993). PROCHECK: a program to check the stereochemical quality of protein structures. J Appl Crystallogr *26*, 283-291.

Leslie, A.W., and Powell, H. (2007). Processing diffraction data with mosflm. In Evolving Methods for Macromolecular Crystallography, R. Read, and J. Sussman, eds. (Springer Netherlands), pp. 41-51.

McCoy, A.J. (2007). Solving structures of protein complexes by molecular replacement with Phaser. Acta Crystallogr D Biol Crystallogr *63*, 32-41.

Murshudov, G.N., Vagin, A.A., and Dodson, E.J. (1997). Refinement of macromolecular structures by the maximum-likelihood method. Acta Crystallogr D Biol Crystallogr *53*, 240-255.

Otwinowski, Z., and Minor, W. (1997). Processing of X-ray diffraction data collected in oscillation mode. In Methods in Enzymology, Charles W. Carter, Jr., ed. (Academic Press), pp. 307-326.

Table S1. Hemagglutination Activity Corresponds to ELISA Measurements, Related to Figure 3. The immunorecognition of foreign cells by tachylectin-2 constructs was measured in an agglutination assay of human type A red blood cells. Hemagglutination was measured in a two-fold dilution series of lysates as well as with purified proteins. The former correlates with the *total binding capacity (*measured by ELISA, also with crude *E. coli* lysates) whereas the latter is a measure of specific activity and correlates with the measurement of binding affinity (K_a). Activities are reported as the minimal concentrations, or greatest dilutions, that gave complete hemagglutination by visual inspection. Positive hemagglutination was observed from lysates expressing WT_V, Anc_{5V}, $AncA_{5B}$ and $AncB_{5B}$ (with high ELISA signals), but was not detected (n.d.) for $WT4_{5V}$, $AncA_{1B}$ or $AncB_{1B}$ (with low ELISA signals). Hemagglutination of the latter set of constructs was detected with purified proteins, demonstrating a small but real binding capacity.

	Lysate (dilution fraction)	Pure protein (nM)
WT_V	0.04 *	20
Anc_{5V}	0.001	0.6
$AncA_{5B}$	0.0001	3
$AncB_{5B}$	0.005	5
$WT4_{5V}$	n.d.	10
$AncA_{1B}$	n.d.	10 (pentamer concentration)
$AncB_{1B}$	n.d.	20 (pentamer concentration)
Null	n.d. (empty plasmid)	n.d. (buffer)

* Incubation with wildtype tachylectin-2 in excess of the minimal agglutination concentration produced agglutination with shorter diameter and dark reddish brown color, suggesting hemolysis.

Table S2. Correlations Matrix of Total Binding Capacity and Biophysical Datasets, Related to Figure 4. Pearson's correlation (r) and the p-value of statistical significance are shown for each dataset comparison.

	Total binding capacity (lysate)	Folding efficiency (purified)	Soluble expression (lysate)	Native stability (purified)	Binding affinity (purified)	Insoluble expression (lysate)
Total binding capacity (lysate)	r = 1<br>p = 0	r = 0.98<br>p < 0.01	r = 0.90<br>$p < 10^{-4}$	r = 0.49<br>p = 0.40	r = 0.71<br>p = 0.18	r = -0.09<br>p = 0.78
Folding efficiency (purified)	r = 0.98<br>p < 0.01	r = 1<br>p = 0	r = 0.95<br>p = 0.01	r = 0.44<br>p = 0.46	r = 0.70<br>p = 0.19	r = 0.72<br>p = 0.17
Soluble expression (lysate)	r = 0.90<br>$p < 10^{-4}$	r = 0.95<br>p = 0.01	r = 1<br>p = 0	r = 0.33<br>p = 0.59	r = 0.61<br>p = 0.27	r = -0.01<br>p = 0.98
Native stability (purified)	r = 0.49<br>p = 0.40	r = 0.44<br>p = 0.46	r = 0.33<br>p = 0.59	r = 1<br>p = 0	r = 0.94<br>p = 0.02	r = 0.92<br>p = 0.02
Binding affinity (purified)	r = 0.71<br>p = 0.18	r = 0.70<br>p = 0.19	r = 0.61<br>p = 0.27	r = 0.94<br>p = 0.02	r = 1<br>p = 0	r = 0.96<br>p = 0.01
Insoluble expression (lysate)	r = -0.09<br>p = 0.78	r = 0.72<br>p = 0.17	r = -0.01<br>p = 0.98	r = 0.92<br>p = 0.02	r = 0.96<br>p = 0.01	r = 1<br>p = 0

Table S3. Trends in Thermodynamic Parameters, Related to Figure 5. The apparent stabilities (C_m) of the native-states and intermediate-states, and cooperativity (m) of folding of the various constructs are in agreement across multiple independent data sets. The stable folding intermediates of WT_V, $AncA_{5B}$ and Anc_{5V} are masked in a two-state representation by a less cooperative native-denatured transition with lower apparent m-value (given multi-state folding, these are not true thermodynamic m-values but provide some basis of comparison). Relative intermediate population is estimated by the numerical integration of curves in **Figure 5D**. Intermediate populations are not applicable (n.a.) to $AncA_{1B}$ and $AncB_{1B}$ due to 2-state behavior.

	Circular dichroism	Unfolding equilibria ($I_{\lambda N}:I_{\lambda D}$)				Unfolding equilibria (I)		
	-GlcNAc	-GlcNAc		+GlcNAc		+GlcNAc		
	Unfolding midpoint (°C)	C_m (M)	m (kcal/mol/M)	C_m (M)	m (kcal/mol/M)	C_{m1} (M)	C_{m2} (M)	Relative intermediate population
WT_V	72	3.1	3.2	5	3.5	4.9	5.2	0.38
$AncA_{5B}$	88	4.8	1.2	5.8	0.8	5.6	6.4	0.92
Anc_{5V}	>90	4.3	2.1	6.6	1.3	6.1	7.3	1.3
$AncA_{1B}$	66	2.1	5.2	3.3	3.4	3.7	n.a.	n.a.
$AncB_{1B}$	65	1.7	5.9	2.5	4.2	2.8	n.a.	n.a.

Table S4. Amino Acid Sequences of Experimental Constructs, Related to Experimental Procedures.

>Anc_{1V} (MPA)
MSPNGTLYGVHGDKFYKGTPPQNDNDNWLARATKIGNGGWNNFKFLFF
>Anc_{1B} (MPA)
MNFKFLFFSPNGTLYGVHGDKFYKGTPPQNDNDNWLARATKIGNGGWN
>Anc_{1B} library (best-performing of round 1)
MSGFKFLFFSPDGTLYGVHNDKFYKGTPPTSDNDNWLARATLIGNGGW
>$AncA_{1B}$
MDGFKFLFFSPDGMLYGVHGDKFYKGTPPTNDNDNWLARATLIGNGGW
>$AncA_{1V}$
MSPDGMLYGVHGDKFYKGTPPTNDNDNWLARATLIGNGGWDGFKFLFF
>$AncB_{1B}$
MSGFKFLFFSPDGTLYGVHNDKLYKGTPPTSDNDNWLARATLIGNGGW
>$AncC_{1B}$
MDGFKFLFFSPDGMLYGVHGDKLYKGTPPTNDNDNWLARATLIGNGGW
>Anc_{5B} (MPA)
M(NFKFLFFSPNGTLYGVHGDKFYKGTPPQNDNDNWLARATKIGNGGWN)$_5$
>Anc_{6B} (MPA)
M(NFKFLFFSPNGTLYGVHGDKFYKGTPPQNDNDNWLARATKIGNGGWN)$_6$
>Anc_{5V} (MPA)
M(SPNGTLYGVHGDKFYKGTPPQNDNDNWLARATKIGNGGWNNFKFLFF)$_5$
>$AncA_{5B}$
M(DGFKFLFFSPDGMLYGVHGDKFYKGTPPTNDNDNWLARATLIGNGGW)$_5$
>$AncA_{5V}$
M(SPDGMLYGVHGDKFYKGTPPTNDNDNWLARATLIGNGGWDGFKFLFF)$_5$
>$AncB_{5B}$
M(SGFKFLFFSPDGTLYGVHNDKLYKGTPPTSDNDNWLARATLIGNGGW)$_5$
>$AncB_{5V}$
M(SPDGTLYGVHNDKLYKGTPPTSDNDNWLARATLIGNGGWSGFKFLFF)$_5$
>$WT4_{1B}$
MDTFKFLFFHPNGYLYAVHGQQFYKALPPVSNQDNWLARATKIGQGGW
>$WT4_{1V}$
MVHPNGYLYAVHGQQFYKALPPVSNQDNWLARATKIGQGGWDTFKFLFF
>$WT4_{5V}$
MV(HPNGYLYAVHGQQFYKALPPVSNQDNWLARATKIGQGGWDTFKFLFF)$_5$
>$WT4_{5V}A$
MVHPNGYLYAVHGQQFYKALPPVTNQDNWLARATKIGQGGWDTFKFLFF
HPNGYLYAVHGQQLYKALPPVSNQDNWLARATKIGQGGWDTFKFLFF
HPNGYLYAVHGQQFYKALPPVSNQDNWLARATKIGQGGWDTFKFLFF
HPNGYLYAVHGQQLYKALPPVSNQDNWLARATKIGQGGWDTFKFLFF
HPNGYLYAVHGQQFYKALPPVSNQDNWLARATKIGQGGWDTFKFLFF
>$WT4_{5V}B$
MVHPNGYLYAVHGQQFYKALPPVTNQDNWLARAAKIGKGGWDSFKFLFF
HPNGYLYAVHGQQLYKALPPVSNQDNWLARATKIGQGGWDTFKFLFF
HPNGYLYAVHGQQFYKALPPVSNQDNWLARATKIGQGGWDTFKFLFF
HPNGYLYAVHGQQLYKALPPESNQDNWLARATKIGQGGWDTFKFLFF
HPNGYLYAVHGQQFYKALPPVSNQDNWLARATKIGQGGWDTFKFMFF
>WT_B
MDDFRFLFFGGESMLRGVYQDKFYQGTYPQNKNDNWLARATLIGKGGW
SNFKFLFLSPGGELYGVLNDKIYKGTPPTHDNDNWMGRAKKIGNGGW
NQFQFLFFDPNGYLYAVSKDKLYKASPPQSDTDNWIARATEIGSGGW
SGFKFLFFHPNGYLYAVHGQQFYKALPPVSNQDNWLARATKIGQGGW
DTFKFLFFSSVGTLFGVQGGKFYEDYPPSYAHDNWLARAKLIGNGGW
>WT_V(*T. tridentatus* tachylectin-2)
MVGGESMLRGVYQDKFYQGTYPQNKNDNWLARATLIGKGGWSNFKFLFL

SPGGELYGVLNDKIYKGTPPTHDNDNWMGRAKKIGNGGWNQFQFLFF
DPNGYLYAVSKDKLYKASPPQSDTDNWIARATEIGSGGWSGFKFLFF
HPNGYLYAVHGQQFYKALPPVSNQDNWLARATKIGQGGWDTFKFLFF
SSVGTLFGVQGGKFYEDYPPSYAHDNWLARAKLIGNGGWDDFRFLFF

Table S5. Crystallographic Statistics, Related to Experimental Procedures. Summary of data collection and refinement statistics for the $AncB_{1B}$ and *N. vectensis* tachylectin-2 crystal structures. * Values in parentheses refer to the data of the corresponding upper resolution shell.

**Data collection**	**$AncB_{1B}$**	***N. vectensis***
Space group	*$P3_112$*	*$P2_12_12_1$*
Cell dimensions:		
a,b,c (Å)	112.2,112.2, 107.3	44.7, 60.9, 88.35
α,β,γ (º)	90, 90, 120	90, 90, 90
No. of copies in a.u.	15	1
Resolution (Å)	30.18-1.65	50.0-1.9
Upper resolution shell (Å)	1.74-1.65	1.93-1.9
Measured reflections	296778	240390
Unique reflections	92166 (13147)*	19656 (946)
Completeness (%)	100.0 (100.0)	99.8 (97.4)
Average I / σ(I)	6.7 (6.4)	29.6 (2.6)
R_{sym} (I) (%)	6.5 (39.4)	4.2 (30.3)
**Refinement**		
Resolution range (Å)	30.12-1.65	50.0-1.9
No. of reflections (I/ σ(I) > 0)	87992	18599
No. of reflections in test set	4637	1002
R-working (%) / R-free (%)	18.6 / 27.7	19.0 / 23.5
No. of protein atoms	5478	1880
No. of ions/ligands atoms	240	
No. of water molecules	500	81
Overall average B factor ($Å^2$)	16.55	20.0
Root mean square deviations:		
- bond length (Å)	0.003	0.024
- bond angle (°)	1.194	1.902
**Ramachandran Plot**		
Most favored (%)	99.3	97.4
Additionally allowed (%)	0.7	2.1
Disallowed (%)	0	0.4

Table S6. **Molecular Clock Divergence in *Frankia* sp. strain EAN1pec, Related to Figure 6.** Uniprot accession codes are labeled with the enumerated data from **Figure 6B**.

	Propeller internal identity (%)	Identity to single motif protein A8LD20 (%)
A8L0X3	62.1	62.5
A8L0Z3	64.0	51.5
A8LFK4	59.5	51.2
A8L1U6	57.3	45.7
A8L579	41.9	42.9
A8LHK5	49.5	42.3
A8L9U4	45.3	40.0
A8KX81	37.1	39.9
A8L1R5	34.1	36.8
A8L653	31.3	32.3
A8L516	30.1	31.2

Figure S1. Ancestral Reconstruction of the Lectin Propeller Motif, Related to Figure 1.
(A) A phylogenetic tree of tachylectin-2 sequence motifs was constructed from an alignment of 10 individual motifs of the *T. tridentatus* and *N. vectensis* tachylectin-2 sequences. Excluding *N. vectensis* motif 5, the motifs are monophyletic with respect to the source protein. The tree topology could not be improved by inclusion of an *X. laevis* outgroup sequence. (B) A probabilistic ancestral motif was reconstructed from the root node of the motif tree in panel B. The posterior probabilities of amino acids given by FastML are plotted as sequence logos in the wildtype Velcro frame and in five additional permutation frames corresponding to an intact structural blade that were tested experimentally. A star (*) indicates the frame from which functional single motifs were obtained.

Figure S2. Correspondence of ELISA Signal to Binding Activity and the Variety of Functional Single Motifs, Related to Figure 3.
(A) To calibrate the non-linear response of ELISA to the functional level of tachylectin-2, raw ELISA signals were measured from a purified wildtype tachylectin-2 concentration gradient. Data were fit to the sigmoidal function $y = \frac{0.843}{1+\left(\frac{5.00}{x}\right)^{0.483}}$

whereby *y* is the measured absorbance and *x* is tachylectin-2 concentration.
(B) Single motifs containing the most probable ancestral substitutions (second round library, p>0.1) were constructed as a mixture of plasmids (~10^4 single motif sequences). This library was transformed into *E. coli* and sampled from the cell lysates of 500 randomly chosen clones. Raw ELISA absorbance was subtracted with the background of an empty plasmid's expression lysate.

Figure S3. Native Propeller Formation and Stability, Related to Figure 3 and Figure 5.
(A) Size exclusion chromatography revealed monodisperse and overlapping elution profiles for tandem fusions and single motif constructs, indicating stable pentamerization of the latter even at low concentration. The upper $AncB_{1B}$ trace is at high concentration (12 μM propeller) and lower traces are at low concentrations (0.8 μM propeller). (B) Proteins showed similar propeller-like signatures by circular dichroism (CD). Shown are native CD spectra measured at 30 ºC. (C) Thermal unfolding was measured by CD (202 nm). In cases of unresolved baselines at high temperature, normalization to zero was assisted by measuring the residual function in ELISA.

Figure S4. Unfolding Parameters by Two-State Fitting, Related to Figure 5. Unfolding equilibria of constructs were again measured by incubating purified protein in GdmCl and measuring fluorescence spectra, as before (**Figure 5C**). However, in order to determine any dependence on the parameterization of curve-fitting, the data in this case were first transformed to two states. At each denaturant concentration, fluorescence intensities at wavelengths corresponding to native and denatured states were taken as a ratio ($I_{\lambda N}$:$I_{\lambda D}$). Two-state unfolding models were fit for single motifs (A) $AncA_{1B}$ and (B) $AncB_{1B}$, identically fused constructs (C) $AncA_{5B}$ and (D) Anc_{5V}, and (E) WT_V, in the presence (filled circles) and absence (empty circles) of GlcNAc. Intermediate populations observed for identical fusions and wildtype (**Figure 5C-D**) manifest here as a less cooperative native-denatured unfolding transition with milder slope (lower pseudo-*m* value; **Table S3**).

Figure S5. Homology-Modeled Structural Domains of Genomic Sequences, Related to Figure 6.
Sequences containing propeller motifs from (A-D) *C. watsonii* and (E-G) *Frankia* sp. strain EAN1pec were modeled with SwissProt using homologs from the pdb. For each panel, the overlap with the pdb sequence is shown by a gray box with the corresponding homology model shown below. (A-B) Single propeller motifs are flanked by domains that do not form propellers, and accordingly, the homology model indicates non-propeller folds. One *C. watsonii* gene is addressed per row. The homology model for the single motif (denoted in green in the sequence diagram, and typically modeled as 4-stranded β-sheet) is shown, alongside the predicted models for the flanking domains. (C-D) Genes composed of six propeller motifs are predicted to form six of the seven blades of a distant propeller homolog, suggesting that they close the radially arranged blades to form intact propellers. (E) An identified *Frankia* gene containing a single propeller motif. (F-G) Genes encoding multi-motif propellers. For all panels, Qmean4 scores (Benkert et al., 2009) given by Swiss-model (Biasini et al., 2014) indicate physical features of model quality and

indicate that the predicted structures are most reliable in core structural regions, as shown by residue coloring using a Qmean4 heat map (blue = low to red = high). Uniprot accession codes are labeled.

Figure S6. Directionality of Emergence in *Frankia* Propeller Motifs, Related to Figure 6.
In a scenario where a single motif was duplicated, fused, and gave a propeller, all propeller repeats would be equally diverged with respect to the single motif and with respect to one another. In the reverse scenario, however, in which a single motif from an exiting propeller was duplicated and inserted into another protein, the propeller repeats would be equally diverged with respect to one another, but the single motif would resemble one repeat more than the others. To test this idea, average identities (black dots, as in **Figure 6B**) were compared against maximum identities (red dots) in a molecular clock plot. Overall, in agreement with the first scenario, the maximum and average identities hold the same trends, indicating that the single motif does not disproportionately resemble one repeat more than the others.

Data File 1. Propeller Motifs in *C. watsonii* and *Frankia* sp. strain EAN1pec, Related to Figure 6.
In *C. watsonii*, two single-motif proteins (Uniprot: Q4BZF5 and Q4BYZ3) each contain 41-42 amino acids that closely correspond to the FG-GAP propeller motif described in Pfam. In the other multi-motif proteins, the core motifs are also extended with sequence that preserves continuous tandem repetition. In *Frankia*, there is one single-motif protein (Uniprot: A8LD20) homologous to the tandemly repeating motifs of many structural propellers of the same organism, and to the single motif proteins of other organisms.
