# Supplementary material for: De Novo Evolutionary Emergence of a Symmetrical Protein Is Shaped by Folding Constraints
Source: Cell. 2016 Jan 28;164(3):476–86. doi: 10.1016/j.cell.2015.12.024 (PMC4735018; doi:10.1016/j.cell.2015.12.024)
Supplement: Data S1. Propeller Motifs in C. Watsonii and Frankia sp. Strain EAN1pec, Related to Figure 6 — In C. watsonii, two single-motif proteins (Uniprot: Q4BZF5 and Q4BYZ3) each contain 41–42 amino acids that closely correspond to the FG-GAP propeller motif described in Pfam. In the other multimotif proteins, the core motifs are also extended with sequence that preserves continuous tandem repetition. In Frankia, there is one single-motif protein (Uniprot: A8LD20) homologous to the tandemly repeating motifs of many structural propellers of the same organism and to the single motif proteins of other organisms. [file mmc2.pdf]

C. watsonii FG-GAP propeller motifs — Extended motifs

```
>Q4BZF5
  20 SGISVSGIGDFNGDNISDLVIGSHQADPNGTS-SGQAHVV   FG------------------------------ 59
>Q4BYZ3
 306 SGRSVSSAGDVNGDGFSDILIGTYHADPNGNSRAGESYVV   FG------------------------------ 347
>Q4BY77
   2 -----SGAGDVNGDGFDDLIIGARSADPNGIGQAGESYVV   FGKAGVFSPSFDLSSLNGSNGFVLNGIDSVDF 68
  69 SGVSVSGAGDVNGDGFDDLIIGANGADPNGNEQAGESYVV   FG------------------------------ 110
>Q4C0A4
  16 ---SNSGAGDINGDGFDDILIGASGADPNGNRSAGESYVI   FGSASGFSGSLDLSSLDGSNGFVLNGIDEFDG 84
  85 SGSSVSGAGDINGDGFDDIIIGAIFADPNGNESAGESYVI   FGS----------------------------- 127
>Q4C0A5
   2 -----SGAGDINGDGFDDIIIGARTADPNGNERAGESYVV   FGAVGGFSGSLDLSSLDGNNGFVINGIDEYDF 68
  69 SGESVSGAGDINGDGFDDILIGAANA-PNGIYDAGESYVV   FGSASGFSASLDLSSLDGSNGFVINGIDEFDF 139
 140 SGESVSGAGDINGDGFDDILIGASSADPNGNYDAGESYVV   FGAASGFSASLELSSLDGSNGFVLNGIDEDDY 211
 212 SGSSVSGAGDINGDGFDDILIGASSADPNGNERAGDSYVV   FGSASGFSASLELSSLDGSNGFVLNGIDEDDF 283
 284 SGNSVSGAGDINGDGFDDIIIGALGASPNGNRSAGESYVV   FGAVGGFTASLELSSLDGSNGFVLNGIDQGDN 355
 356 LGVSVSGAGDINGDGFDDIIIGALGASPNGNRLAGESYVI   FGS----------------------------- 398
>Q4BWI7
  75 FGRSVSNAGDVNGDGIDDLIIGAEGADPNGNSYAGETYVV   FGSES-FGDTLNLSSLNGSNGFVINGIDARDY 145
 146 SGRSVSNAGDINGDGIDDIIIGAFLADRNGNSLLGQTYVV   FGSES-FDSTLNLSSLDGSNGFVISGIDARDY 216
 217 SGRSVSNAGDVNGDGIDDFIIGAYGAEPNGNRFAGETYVI   FGSES-FGNTLNLSSLDGNNGFVINGIDEFDN 287
 288 SGFSVSNAGDINGDGFDDLIIGAFLAQPNGNIDAGETYVV   FGSSS-FEDTLNLSSLDGSNGFVINGIDEFDN 358
 359 SGFSVSNAGDINGDGFDDLIIGAFFADPNGNEDAGETYVI   FGSES-FGSTLNLSSLNGNNGFVINGIDAGDN 429
 430 SGFSVSNAGDVNGDGIDDLIIGADPAEANGNSYAGQTYVI   FGGQNNNSNSL-LS------------------ 482
```

<u>*Frankia* sp. strain EAN1pec, WD40 propeller motifs</u>

```
>A8LD20
---SLS----GSPLRGHTDS----VRSVAFSPDGRTLASPSQDRTV--------RLWD
>A8L0Z3
VTSSTNSRTLGAPLAGHTST----VRAVAFSPDGRILASASDDEPV--------RLWD
VTDPGDARPLDASLTGHSGW----VHSVAFSPDGHTLASAGDDHTV--------RLWN
VTDPANAHPLGAPLTGHTST----VWAVAFSPDGRILASAGNDETV--------TLWD
VADPAQARPLDVISETR------AVRSVAFSPDGRILASAGDDGTA--------SLWN
VADPTNPRPLGTPLAGHTNT----VWVVAFSPNGHTLASAGDDHTV--------RLWN
VTDPANAHPLGAPLTGHTST----VRSVAFSSDSRTLASGSDDHTV--------RLWD
VIDPANAHPRGASLTGHSSW----VRSVAFAPDGRTLASGSDDHTM--------RLWD
>A8L0X3
---SLS----GSPLRDHTDS----VRSVAFSRDGRTLASASQDGTA--------RLWD
IAERTSQP-LTGRIA---------VWSVAFSPDKHTLASANGDSTV--------QLWD
VAEGTLPH-PVASLPGHSDA----VGSVAFSPDGRTLASASDDHTV--------RLWD
VATGTT----THTLTDHTGP----VNSVAFSRDGRTLASASDDHTV--------RLWD
VAEGTL----LRTLPGHTEP----VMSVAFSPDRRTLASASQDNTV--------RLWD
VAARTAPR-LVGSLSDHTHW----VMSVAFSPDGRILASASQDRTV--------RLWD
VAARTT----THTLTGHTGP----VFSVAFSLDGRTLASASDDNTV--------RLWD
>A8LFK4
PAAQAL----GQPLTDHTDW----VASVAFSPDGHTLASSSKDTTV--------RLWD
ITDRTRPHPLGQPLAGHTLG----VMSVAFSPDGNTLASSSRDTTI--------RLWD
ITDRTRPHPLGQPLTGHTDA----VTSVAFFSDGRTLASSSRDTTI--------RLWD
ITDRTRTRPLGSPLSGHSDW----VTSLALTMDGRTLASSSLDSTV--------RLWN
MADRSHPQPIGLPLTGHTGG----VNSVAFSLDSRTLASSGRDTTI--------RLWD
VTDRSTPRLLGAPITGHANT----VGPLTFSQDGDTLVSGSYDDTV--------RIWD
VTDRSHPRLLGLPLTGHTDW----IWSVALSPDGQTLASGSKDNTI--------RLWA
>A8L1U6
VTDRTRPTPLEKTLYGH-TG---WVRSVVFSPDGRTLASGSDDDTV--------RFWD
MSDPTDPTPLGKPLTGH-TDP---IAAVAYSPDGRTLATSSTDRTV--------RFWD
MSDRADPTPLGKPLTRHSTDPTSWVAAMAYSPDGRTLATGGLDNTL--------RLWD
VTDRTNPTPLGPPLTGH-TKT---VAFLAYSPDGRTLATGSYDRTV--------RLWD
VSDRTSPAPLGPPLTGHTNP----LMSLAYSPDGRTLATSASDYST--------RFWD
VSDRTRPTPLGEPIFLGDSPSVWWVVGLAYSPDGHTLATISGDRLV--------RFWD
>A8L579
IGRRAAARPAGPPLAGHGGW----VLSADFSPDGMIMATSAKDGEV--------LLWD
VAEPAAPRLVGRTPDGPDDG----VTSVVFSPDGHTLAGTSWDGSL--------RLWD
VTRPGAPHLLGRPLRAHAGP----VRSAAFATDGRMLATGSDDRSV--------QLWD
MADRGAPRPLGGPVTGATSF----VTSVAFAPDDHLLVAAGYDRDV--------LFWD
ITNRRAPRRLAR-IVAGPTS----ALVAAFAPDGRTLATGGVDGVI--------RLWD
VTVPDRPTALGRPLAGHDNR----VWALAFAPDGRTLASGGFDNVV--------RLWD
TSDRTRARPRGRPLAGHADW----VMSVRFAPHGRILASTGKDQTI--------RLWS
>A8LHK5
-SRNVEVTTRVALTG-HERD----VTSAAFSPDGRLLATTSKDGT---------RLWD
---TTTGRTVGRLSGRKISA----VHGCAFSPDGDLLATTGSDKTA--------RIWE
----IATERLALTLAGHKGP----VYGCAFSPDGRLLATVSTDRTV--------KLWG
---VSTGTNIATLTG-HRGS----VYGCAFSPDGRLLVTAGAEST---------LLWD
---VTIGETITSLAG-HTNF----ANGCSFSPDGLLLATTSNDGT---------RLTD
----TPTGTTTLTLPGS-------AQSCAFSPDGVLLATASTDDTA--------RLWD
---VATGTAVATLTG-HSST----VMACAFAPYGLLLATTSTDKTA--------RLWD
>A8L9U4
-LRTSYSAATATVLTGHTGS----VLGLGISPDGRTIATSGADNVA--------RLWD
VSDRTRPRQLSTIDAHGAW-----VLDAAFSPDGKLLATVGYDRSA--------RLWD
IGDRTRPKQLSSMLAHNGY-----VLDAAFSPDGRMLATSGYDNTA--------RLWD
ITDPRQPHELAVLDRHTSW-----VNEVAFSPDGKLLATASADHTA--------RLWD
IANPRQPRPLAA-ITTHTDF----VWTVAFSPDGRRLATGAYDGLV--------KIWD
```

```
ITDPTRPGATASFRADEKW-----VFDVAYSPDGRTLATAGWDTSV--------HLWD
>A8KX81
LAREPGVRPLGRPLADQAGW----ALSTAFSPDGKVMASSSRKGGV--------WLWN
LAVPEDPHPIGRPLTGHTNR----VWSLAFSPDGGTLASSGFDNSV--------RLWD
ITDSGASKPAGRALTDDSGP----IWSVAFSADGRTLASGSDDTTV--------RLWD
MADPATPVRIDPALTGPRDG----VTSLAFSPDGSLLAGGSWDGSI--------WLWD
MADRGAPKRLAQ-SLSTPAT----TYVAAFSPNGRLLATGSTDGLV--------RLWD
MTNRARPWQFVR-LSSDMEF----VTSVAFSADNRLLVAAGFSRTI--------AIWD
>A8L1R5
GLFAADVDPVVAPLGSR-------VLSAALRPDGRLLAAGTEAGTI--------ELWD
LTDLAHPVHAGTISGVGD-----WVYSVAFNPGGNLLAAGVGDGAV--------RLWN
VTDPARAGALATIAFHRD-----RVRSVAFAPDGGTLASGGDDGQV--------GLWA
VTDPSHPQRRSATDGAVA-----GIRSLAFSPRGGLLALAGNDGSV--------RLWN
VADPARPATSSTLRGTGR-----TVQSVAFSADSSTLAAGGIDGSV--------HTWR
VDGPGSVVDLGSTPGGVGG-----VTSVGFSPEGAILVSASEDETV--------RLTD
ISAPADPVLLTDLRGHTKA-----VSAAMFVPGGRTVVSASGDGSV--------RLWT
>A8L653
VRDPAKPSRISDVPGPAGG-----TAELLFSPDGRTLALGGLNSPPEPAFQARVETWD
VTDLSRPRRLASLIAHRSS-----VRDLTFSPDGRTLVSAA-ERSV--------KLWD
VTDPRRLRLVSELPEFPGG-----VWEVRFSPDGRTLAAGGANPFA--------TLWD
-VTRMDAPRQIADLPGHSAS----VTSVAFSPDGTQLATGSNDNTV--------RIWD
VTEHDSPTLIEKLARSAGSEAG--IEEILYTRDGEKLVGVIFTVPA--------VVWD
>A8L516
ITDPRRPFMITAFEGPAGG-----VFSLRFSPDGRTLVLGGLGSSPAPAFSASLETWD
VRDPRHPARLASTVAHRGS-----VRDIQFSEDGHVMASVA-DRTV--------QLWD
VSDPRRIAARATLPDFPGA-----ALRAAFAPDGRTLAVGGGGPYA--------TLWN
ITDPARPTRTASLPGHVSE-----VNTVTFSPDGRTLVTGSGDNSV--------RVWD
VRRPSNPRLVERLSRSAGTNSG--IGTVAFSRDGSTLVGVVFTEPG--------ALWD
```

Single-motif proteins homologous to the *Frankia* series

Genbank ID	Length	Organism
297156412	73	Actinobacteria; Streptomyces bingchenggensis BCW-1
357265231	83	Cyanobacteria; Crocosphaera watsonii WH 0003
357265846	99	Cyanobacteria; Crocosphaera watsonii WH 0003
493214827	58	Cyanobacteria; Nodularia spumigena
497319093	71	Cyanobacteria; Synechocystis sp. PCC 7509
499925710	68	Actinobacteria; Frankia alni
518321537	72	Cyanobacteria; Calothrix sp. PCC 7103
518334279	105	Cyanobacteria; Pleurocapsa sp. PCC 7319
605047033	147	Cyanobacteria; Richelia intracellularis
651290729	77	Actinobacteria; Actinokineospora inagensis
658299017	107	Cyanobacteria; Planktothrix agardhii NIVA-CYA 126/8
664265184	79	Actinobacteria; Streptomyces sp. NRRL S-337
737391083	98	Cyanobacteria; Dolichospermum circinale
739354603	164	Cyanobacteria; Richelia intracellularis
746136515	167	Cyanobacteria; Aphanizomenon flos-aquae
750668271	67	Cyanobacteria; Prochloron didemni
750668722	67	Cyanobacteria; Prochloron didemni
752584810	84	Actinobacteria; Frankia sp. EAN1pec
752686059	66	Chloroflexi; Roseiflexus sp. RS-1
808671128	93	Actinobacteria; Mycobacterium conceptionense
